# Abortion stigma among abortion providers in high-income countries: a mixed methods systematic review

**DOI:** 10.1080/26410397.2026.2668884

**Published:** 2026-05-22

**Authors:** Marie Bernard, Jana Niemann, Dennis Jepsen, Nadja Freymüller, Laura Weinhold, Céline Miani, Claudia Luck-Sikorski

**Affiliations:** a Research Associate, Institute of Medical Sociology (IMS), Medical Faculty, Martin Luther University Halle-Wittenberg, Halle-Wittenberg, Germany.; d Research Associate, Department of Sustainable Environmental Health Sciences, Medical School OWL, Bielefeld University, Bielefeld, Germany; Research associate, Institute for Rehabilitation Medicine, Medical Faculty, Martin Luther University Halle-Wittenberg, Halle-Wittenberg, Germany; e Research Assistant, Institute of Medical Sociology (IMS), Medical Faculty, Martin Luther University Halle-Wittenberg, Halle-Wittenberg, Germany; f Professor of Social and Gender Epidemiology, Department of Epidemiology and International Public Health, School of Public Health, Bielefeld University, Bielefeld, Germany; g Professor of Mental Health and Psychotherapy, SRH University of Applied Sciences Heidelberg, Gera, Germany

**Keywords:** abortion, pregnancy termination, stigma, healthcare providers, reproductive health, systematic review

## Abstract

Negative attitudes towards abortion affect providers who perform abortions and lead to discrimination and emotional distress. This review examines the prevalence, forms, and consequences of felt abortion stigma among providers in high-income countries. A mixed-methods systematic review was conducted to update a 2016 review on abortion stigma. Following the PRISMA guidelines, searches in MEDLINE, CINAHL, PsychINFO, LIVIVO, and the Cochrane Library identified peer-reviewed studies published after March 2015. Qualitative, quantitative, and mixed-method studies were included. Data extraction and synthesis followed JBI guidelines. Twenty-eight studies were included (23 qualitative, 4 quantitative, 1 mixed-methods). Quantitative findings showed that abortion-related stigma varies by legal and sociocultural context and is higher in restrictive settings. Greater felt stigma was associated with burnout, emotional exhaustion, and job strain. Qualitative synthesis identified three main themes: (1) institutional and structural stigma linked to legal restrictions, limited training, and professional isolation; (2) individual-level stigma, including harassment, social exclusion, and concerns about disclosure; and (3) mitigating factors, such as legal reforms (e.g. decriminalisation) and provider advocacy for reproductive rights through patient empowerment and respectful, balanced communication. Abortion stigma at the individual level is strongly shaped by institutional and structural contexts, underscoring the need for interventions beyond individual coping strategies. Efforts to reduce abortion stigma should address not only personal attitudes but also the broader social, cultural, and legal frameworks that sustain it. Healthcare systems are critical intervention points, as clinical practices and gatekeeping can reinforce stigma, while reforms in training and policy can help challenge it. *DOI: 10.1080/ 26410397.2026.2668884*

**Trial registration number:**

https://doi.org/10.17605/OSF.IO/2XPJD.

## Plain language summary

Abortion is a common medical procedure but people who provide abortion care often experience stigma. This means that they may face discrimination, social isolation, or emotional distress simply because of their work. Stigma can also reduce the number of healthcare professionals willing to provide abortion care. This can lead to reduced access to safe abortions, particularly in places where abortions remain controversial. Although numerous recent studies have explored this topic, a comprehensive, updated review is lacking. We conducted a systematic review of 28 quantitative and qualitative studies to gain a full picture of the issue. Our goal was to examine how abortion providers in high-income countries experience stigma and how much it affects them. Quantitative studies have found that stigma levels vary depending on the country and professional role. Providers in countries with restrictive abortion laws reported the highest stigma and those in more liberal settings experienced less stigma. Stigma was linked to negative effects such as emotional exhaustion, job strain, and burnout. Qualitative studies have focused on personal experiences and highlighted how stigma affects daily life. Many providers described feeling judged, isolated, or even harassed. Some providers kept their work secret to avoid negative reactions. This study highlights the need for legal protection, workplace support, and public awareness campaigns to reduce abortion stigma.

## Résumé

Les attitudes négatives à l’égard de l’avortement touchent les professionnels de santé qui le pratiquent et entraînent discrimination et détresse psychologique. Cette étude examine la prévalence, les formes et les conséquences de la stigmatisation de l’avortement ressentie par ces professionnels dans les pays à revenu élevé. Un examen systématique à méthodes mixtes a été mené afin de mettre à jour une revue de 2016 sur la stigmatisation de l’avortement. Conformément aux lignes directrices PRISMA, les recherches dans MEDLINE, CINAHL, PsychINFO, LIVIVO et Cochrane Library ont permis d’identifier des études évaluées par des pairs et publiées après mars 2015. Des études qualitatives, quantitatives et à méthode mixte ont été incluses. L’extraction et la synthèse des données ont suivi les directives JBI. Vingt-huit études ont été retenues (23 qualitatives, 4 quantitatives et 1 à méthode mixte). Les résultats quantitatifs ont montré que la stigmatisation liée à l’avortement varie selon le contexte juridique et socioculturel et qu’elle est plus prononcée dans les contextes restrictifs. Une stigmatisation plus aiguë était associée au burn-out, à l’épuisement psychologique et à des tensions au travail. La synthèse qualitative a identifié trois thèmes principaux : (1) la stigmatisation institutionnelle et structurelle liée aux restrictions légales, à une formation limitée et à l’isolement professionnel ; (2) la stigmatisation individuelle, notamment le harcèlement, l’exclusion sociale et les craintes liées à la divulgation ; et (3) les facteurs d’atténuation, tels que les réformes juridiques (par exemple la dépénalisation de l’avortement) et le plaidoyer des professionnels de santé en faveur des droits reproductifs par l’autonomisation des patientes et une communication respectueuse et équilibrée. La stigmatisation de l’avortement au niveau individuel est fortement influencée par les contextes institutionnels et structurels, soulignant la nécessité d’interventions allant au-delà des stratégies d’adaptation individuelles. Les activités destinées à réduire cette stigmatisation doivent prendre en compte non seulement les attitudes personnelles, mais aussi les cadres sociaux, culturels et juridiques plus larges qui la perpétuent. Les systèmes de santé représentent des points d’intervention essentiels, car les pratiques cliniques et le contrôle de l’accès aux soins peuvent renforcer la stigmatisation, alors que les réformes de la formation et des politiques peuvent contribuer à la combattre.

## Resumen

Las actitudes negativas hacia el aborto afectan a los prestadores de servicios que realizan abortos y propician discriminación y angustia emocional. Esta revisión examina la prevalencia, las formas y las consecuencias del estigma del aborto sentido por los prestadores de servicios en países de altos ingresos. Se realizó una revisión sistemática de métodos mixtos para actualizar la revisión de 2016 sobre el estigma del aborto. Siguiendo las directrices de PRISMA, las búsquedas en MEDLINE, CINAHL, PsychINFO, LIVIVO y la Biblioteca Cochrane identificaron estudios revisados por pares publicados después de marzo de 2015. Se incluyeron estudios cualitativos, cuantitativos y de métodos mixtos. La extracción y síntesis de datos siguieron las directrices de JBI. Se incluyeron 28 estudios (23 cualitativos, 4 cuantitativos, 1 de métodos mixtos). Los hallazgos cuantitativos mostraron que el estigma relacionado con el aborto varía por contexto legislativo y sociocultural y es más alto en entornos restrictivos. Un mayor estigma sentido estaba asociado con agotamiento, desgaste emocional y tensión laboral. La síntesis cualitativa identificó tres temas principales: (1) estigma institucional y estructural vinculado con restricciones legislativas, formación limitada y aislamiento profesional; (2) estigma a nivel individual, que abarca acoso, exclusión social y preocupaciones sobre divulgación; y (3) factores mitigantes, tales como reformas legislativas (p. ej., despenalización) e incidencia política por parte de los prestadores de servicios a favor de los derechos reproductivos por medio del empoderamiento de las pacientes y de una comunicación respetuosa y equilibrada. El estigma del aborto a nivel individual es moldeado en gran medida por los contextos institucionales y estructurales, lo que subraya la necesidad de realizar intervenciones más allá de las estrategias de afrontamiento individual. Los esfuerzos para reducir el estigma del aborto deben abordar no solo las actitudes personales sino también los marcos sociales, culturales y legislativos que lo sustentan. Los sistemas de salud son puntos de intervención críticos, ya que las prácticas clínicas y el control de acceso pueden reforzar el estigma, mientras que las reformas en formación y políticas pueden contribuir a combatirlo.

## Introduction

Despite being a common process in healthcare, abortion care remains highly stigmatised.^1^ Abortion providers^†^ worldwide face widespread stigma and emotional challenges related to their work,^2^ which often discourages open discussions about their profession.^3^ This disclosure in public and private discourse reinforces the perception that abortion care is uncommon or deviant,^3^ potentially limiting the number of professionals willing to offer essential services from the perspective of sexual and reproductive rights. Abortion providers occupy a pivotal position at the intersection of individual, institutional, and structural stigma, navigating stigmatised interpersonal encounters while delivering care within healthcare systems and legal frameworks that regulate and constrain abortion provision.^2^ This can undermine providers’ professional standing, isolate them from their peers, and expose them to harassment or discrimination.^4^ Consequently, abortion-related stigma is one of the main barriers to providing and obtaining safe abortion care services.^5^ In 2016, Hanschmidt et al.^6^ investigated to what extent abortion providers are impacted by stigma. They were able to review one quantitative study^7,8^ and two qualitative studies^9,10^ were included in the analysis. While this systematic review^6^ provided valuable insights into the stigma experienced by abortion providers, a considerable amount of new research has been published since that time, warranting an updated synthesis.

Abortion stigma manifests differently for abortion providers than for abortion seekers. While patients experience abortion as a singular event, providers remain continuously involved in abortion care, embedding it as a fundamental aspect of their professional identity.^11^ Providing abortion services is often considered “*dirty work*”^9,10,12,13^ and abortion providers might be seen as “*dirty workers*”*.*^2^ Consequently, abortion providers could become targets for stigmatisation. There is evidence that abortion providers face individual stigma, as they are often associated with negative stereotypes such as being callous, immoral, or less competent than other healthcare professionals.^3,11^ Consequently, they frequently become targets of harassment and discrimination.^10^ Simultaneously, abortion providers may act as sources of stigma,^6^ explicitly or implicitly reproducing stigmatising attitudes through judgmental communication, gatekeeping practices, providing false information that exaggerates abortion-related health risks, or discouraging counselling, which can deter individuals from seeking or continuing abortion care and constitute an additional barrier to access.^6^

In the following section, we outline the theoretical foundations of this perspective review by describing three distinct levels of abortion stigma: individual, institutional, and structural. The conceptual foundation of this review is rooted in the understanding that abortion stigma is not merely a personal or interpersonal phenomenon but a socially constructed system of power and exclusion.

## Conceptual framework: the individual, institutional, and structural levels of abortion stigma

In 2009, Kumar, Hessini, and Mitchell^14^ were the first to define abortion-related stigma as a “negative attribute ascribed to women who seek to terminate a pregnancy that marks them, internally or externally, as inferior to ideals of womanhood.” (p. 628). This definition draws on Goffman’s understanding of stigma as an attribute that profoundly discredits individuals^15^ and was explicitly centred on people seeking abortion care. Building on this foundational conceptualisation, Norris et al.^11^ later broadened the analytical scope of abortion stigma by demonstrating that stigma extends beyond abortion seekers to encompass other groups associated with abortion, including abortion providers. By situating abortion stigma across multiple social positions, Norris et al.^11^ highlighted that abortion-related stigma is not confined to those undergoing the procedure but is also experienced interpersonally by those involved in its provision.

**Figure 1.**
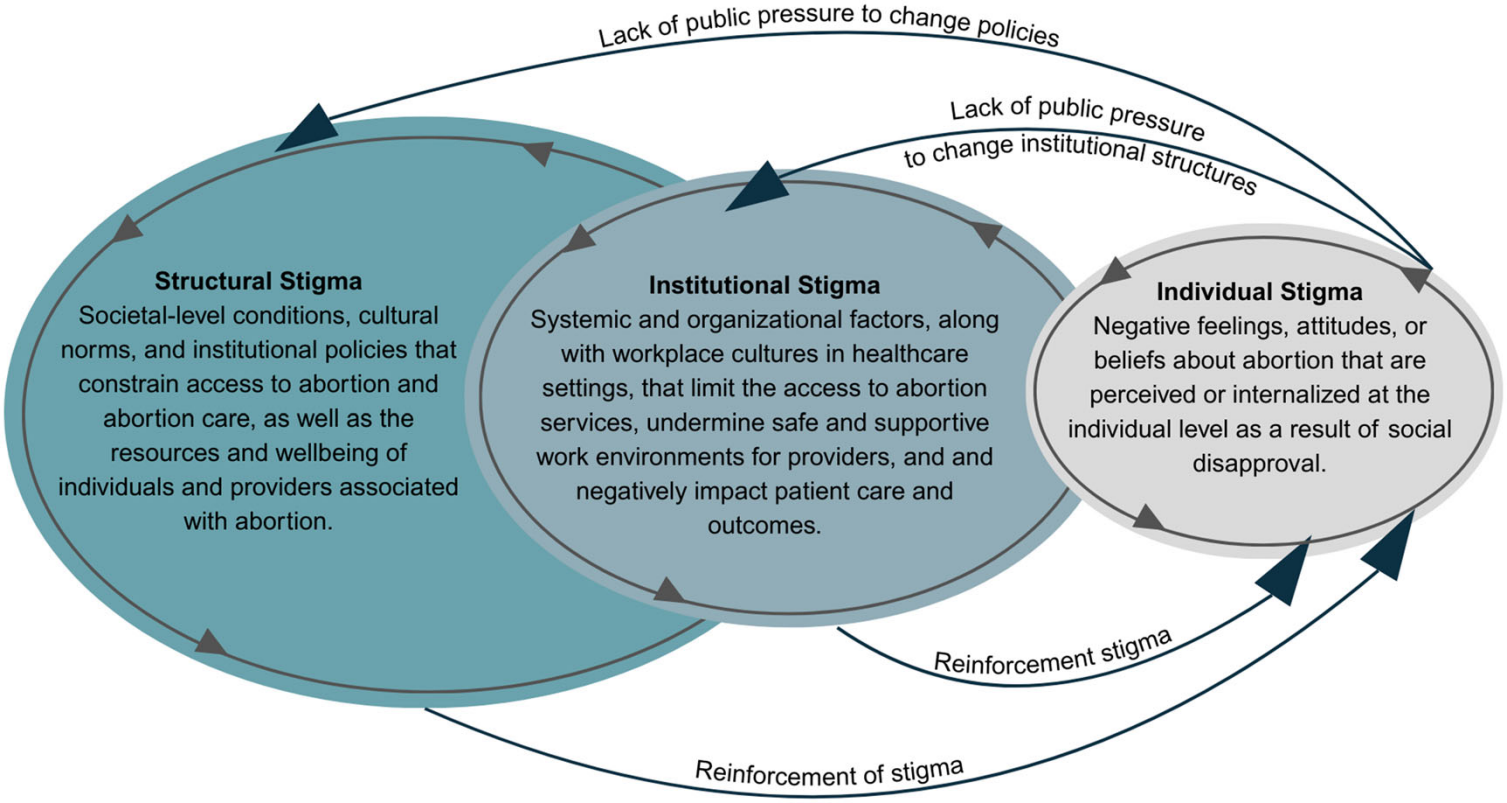
Theoretical framework of a continuum of individual, institutional, and structural forms of stigma. Individual,^17,18^ institutional,^17,18^ and structural^18,19^ forms of stigma exist on a continuum and often overlap, with each level reinforcing the others. Importantly, when stigma is embedded in structural conditions such as laws, policies, and cultural norms, individuals have less agency to challenge or resist it on their own

Abortion-related stigma operates across multiple, interrelated levels, including the micro level (inter- and intra-individual stigma), the meso level (institutional stigma), and the macro level (structural stigma), with each level reinforcing and shaping the others.^11,14,16^ An overview of this multilevel conceptualisation is provided in Figure 1 and elaborated upon in the following sections.

On the micro-level,^4^ **individual stigma** – often referred to as felt stigma^20^ – encompasses individuals’ perceptions of negative feelings, attitudes, or beliefs directed at them because of socially disapproved or marginalised characteristics, behaviours, or experiences.^21^ For those who recognise negative perceptions from the outside, it can lead to internalised shame, self-blame, or unworthiness, resulting in isolation or, in the case of abortion providers, to offer abortion care based on the anticipation of stigma.^4,22^ Hanschmidt et al.^6^ reported that at the individual level, providers often managed stigma by concealing their work, although most still felt proud of their roles.

While research on individual stigma is valuable, it is important to note that stigmatising behaviours are embedded within broader social contexts.^14^ Abortion-related stigma should therefore not be understood merely as an individual or community-level phenomenon, but rather as a socially constructed mechanism rooted in wider systems of inequality and injustice.^1,4^ Numerous scholars have highlighted the central role of institutional and structural dimensions of stigma, as well as power relations,^1,4,23–26^ which both shape and are shaped by individual-level stigma.

Institutional and structural stigmas are sometimes used interchangeably,^23,27,28^ this reflects their deep interconnectedness.^17^ However, similar to other forms of discrimination such as racism,^18^ we argue that maintaining a conceptual distinction between the two is analytically valuable. Distinguishing between institutional and structural stigma provides a clearer understanding of how stigma operates within organisations versus its embedding in legal, political, and cultural systems, ultimately enabling more precise and effective intervention. Expanding on this differentiation, we focus on structural stigma, which functions on a larger societal scale and influences the institutional settings in which abortion services are provided.

**Institutional stigma** is embedded in healthcare settings’ systems, norms, and organisational structures.^14^ Our definition refers to the systemic and organisational factors as well as workplace cultures within the healthcare systems that contribute to the stigmatisation of abortion and those who provide it. This form of stigma is evident in the separation of abortion services from broader healthcare provision, permitting conscientious objection as well as exclusion from medical curricula^4,14^ – an institutional dynamic that can hinder access to comprehensive care. Institutional abortion stigma plays a significant role. In the United States, for example, abortion services are commonly provided in standalone clinics, and providers are often marginalised from mainstream healthcare. In their systematic review, Hanschmidt et al.^6^ found that workplace stigma was reflected in dismissive attitudes from patients and colleagues; however, many providers simultaneously reported supportive and solidaristic team environments.

**Structural stigma** can be defined as “*societal-level conditions, cultural norms, and institutional policies that constrain the opportunities, resources, and wellbeing of the stigmatized”* (p. 2).^19^ A structural view of stigma highlights its presence across interconnected layers of society, including cultural norms, institutional frameworks, legal systems, public narratives, and personal interactions.^1,14,16^ Abortion-related stigma at the macro level is expressed by decision- and law-makers to avoid and neglect the topic, such as the exclusion of abortion from national health policies or mandatory waiting periods.^4,16^ Additionally, a persistent lack of comprehensive training for abortion providers is further exacerbated by legal restrictions and institutional policies, limiting their ability to offer or provide abortion care.^2,11,16^ According to Hanschmidt et al.,^6^ abortion providers frequently experienced marginalisation shaped by public discourse and political narratives, with more than half reporting stigma from the broader medical profession and society.^7,8^

A comprehensive understanding of institutional and structural abortion stigma requires integrating both its roots, such as discriminatory laws, restrictive policies, and entrenched power imbalances, and its manifestations, including the exclusion of abortion training from medical curricula, insufficient institutional support for providers, and the marginalisation of abortion services within healthcare systems.^4^ These dynamics are particularly consequential as they undermine international human rights obligations to ensure non-discriminatory, stigma-free access to abortion care.^29^

## Objectives and research questions

Given these developments and persistent barriers, we aim to update the systematic review conducted by Hanschmidt et al.,^6^ offering a current synthesis of evidence on the experiences of abortion care providers in high-income countries (HICs). As outlined in our study protocol^30^, there are substantial differences in the availability of legal abortion between HICs and low- and middle-income countries (LMICs).^31^ In general, individuals in HICs have better access to comprehensive sexual and reproductive healthcare, including abortion services.^32^ Unsafe abortions are more common in LMICs and can strongly shape stigma. To allow for more consistent and comparable insights into how providers experience abortion stigma, we chose to focus our analysis exclusively on HICs.

We conducted a systematic review using a mixed-methods approach, focusing on the subjective experiences of abortion stigma among abortion providers in HICs. Therefore, we aimed to answer the following research questions:

**RQ1:** What definition(s) did the studies employ for their theoretical conceptualisation of abortion-related stigma?

**RQ2:** To what extent do abortion providers in HICs experience abortion-related stigma from a quantitative perspective?

**RQ3:** What factors are associated with stigma towards abortion providers from a quantitative perspective?

**RQ4:** What are the lived experiences concerning stigma among abortion providers in HICs from a qualitative perspective?

## Methods

This systematic review was conducted according to the PRISMA (Preferred Reporting Items for Systematic reviews and Meta-Analyses) guidelines^33^ (see Supplementary Table 1) and the Joanna Briggs Institute (JBI) guidelines for mixed-method systematic reviews.^34^

### Inclusion criteria

*Participants:* This systematic review included studies on abortion-related stigma among abortion providers (i.e. practitioners, midwives, nurses, obstetricians, gynaecologists, or doulas working in abortion care).

*Phenomena of interest:* We examined research that investigated the magnitude of abortion stigma (prevalence or covariate) and how abortion providers experience this stigma.

*Context:* We only included studies about HICs. We established the HIC status by identifying the location of the study and adhering to the World Bank definition^35^.

*Types of studies:* Our selection encompassed peer-reviewed studies that employed various research methodologies, including quantitative, qualitative, and mixed-methods approaches. Quantitative studies comprised both clinical and non-clinical investigations. Qualitative studies incorporated, but were not limited to, in-depth interviews, group discussions, analyses of (social) media, and autobiographical works.

### Search strategy

We conducted a systematic literature search from January to February 2023, updated on February 27, 2024, focusing on peer-reviewed studies published in English that examined abortion stigma in HICs using qualitative, quantitative, and mixed methodologies. The research databases included MEDLINE, CINHAL, PsychINFO (via EBSCOhost), LIVIVO, and Cochrane Library. Two independent reviewers (JN and MB) searched using keywords from a previous systematic review^1^: [abortion OR termination of pregnancy OR voluntary interruption of pregnancy] AND [stigma* OR discrimination*]. The search was limited to titles and abstracts (see Supplementary Table 2 and the previously published study protocol^30^). The review was based on Hanschmidt et al.,^6^ including only studies published after March 2015.

### Study selection

**Figure 2.**
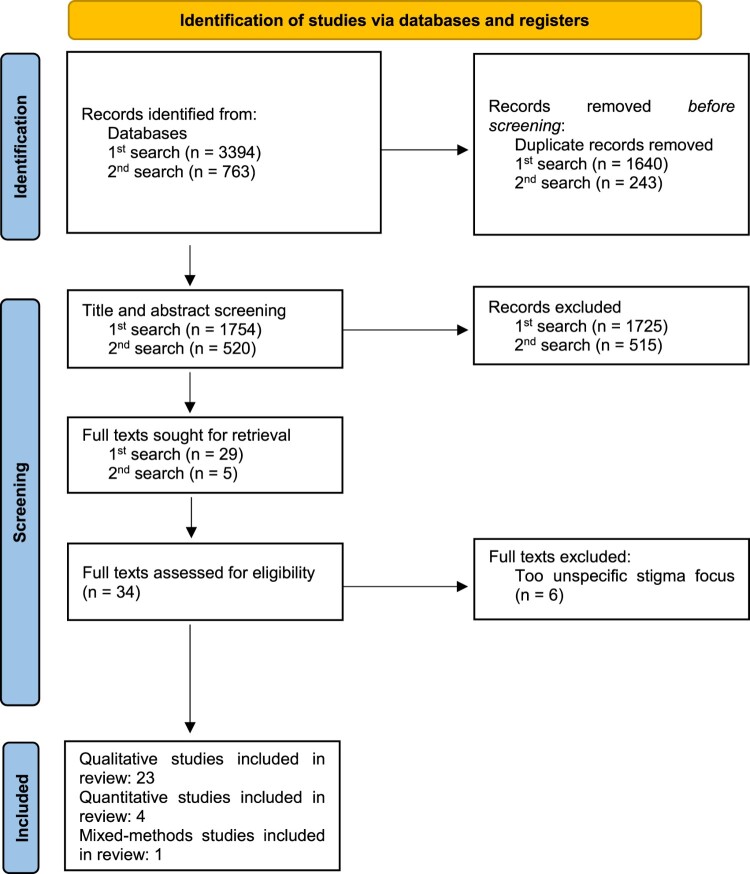
PRISMA flow diagram of the selection process

We imported the identified publications into the software Rayyan^36^ and removed duplicates. Two researchers (JN and MB) independently screened titles and abstracts for eligibility according to the inclusion criteria. JN and MB moved eligible studies to Excel for a detailed full-text review conducted independently. If the full text could not be accessed, the correspondence authors were contacted. For the current publication, all full texts were retrieved. Disagreements during the selection were resolved through discussion. The stages and outcomes of the search and selection processes are illustrated in Figure 2.

This figure illustrates the study-selection process. The initial search was conducted between January and February 2023 and updated on February 27, 2024.

### Critical appraisal

#### Quantitative studies

DJ, JN, and MB evaluated the methodological quality of the quantitative studies using the Risk Of Bias In Non-randomised Studies – of Exposure (ROBINS-E) tool.^37^ The researchers settled any conflicts that emerged during the assessment through discussion.

#### Qualitative studies

JN and LW used the JBI Qualitative Assessment and Review Instrument for interpretive and critical research^38^ to establish dependability in the qualitative research synthesis. JN critically evaluated qualitative studies to assess their methodological rigour. To ensure rigour, an additional reviewer (LW) assessed 20% of the included studies. Inter-rater reliability was calculated using Krippen-Dorff's alpha coefficient,^39^ resulting in a score of 0.945. Any assessment discrepancies were resolved through discussion until mutual agreement was reached.

### Data extraction and synthesis

Following the JBI guidelines for mixed methods systematic reviews,^34^ we chose a convergent separate approach to synthesise quantitative and qualitative data from the studies. Therefore, we analysed the quantitative and qualitative data separately and integrated them. Data were extracted by coding using MAXQDA 2024 for both strands of research.

#### Quantitative studies

DJ, JN, and MB gathered data on the article's definition of abortion stigma, its role as an outcome or predictor variable, geographic location, sample size, validity, reliability, prevalence, and its relationship with the independent variables. Due to diverse measurement approaches in quantitative studies, the team opted for a narrative synthesis,^34^ including a mean value comparison of the Abortion Provider Stigma Survey instrument (APSS), instead of a meta-analysis, conducted by MB.

#### Qualitative studies

We followed the JBI meta-aggregation approach for qualitative studies.^38^ JN extracted the data using an adapted JBI data extraction tool^40^ tailored to the study's phenomenon of interest. JN assessed credibility, categorising the data as unequivocal, credible, or not. JN and NF coded data based on our theoretical framework. We assessed confidence in abortion providers through qualitative research synthesis, using the ConQual approach, with dependability determined by the JBI critical appraisal tool and credibility determined by the quality and detail of findings.^41^ JN, NF, DJ, and MB validated the appraisal, extraction, and synthesis of the findings.

#### Integration of quantitative and qualitative evidence

After separately analysing the quantitative and qualitative data, we used configurative analysis to merge the evidence by comparing the synthesised findings from both approaches for compatibility. Adhering to the JBI mixed-method systematic review guidelines,^34^ JN and MB conducted the integration.

### Reflexivity statements

As an author team, we approach abortion as a fundamental sexual and reproductive health right and consider it essential for upholding human dignity. We understand abortion stigma as a significant barrier to realising this right and achieving universal access to health care. In conducting this review, we engaged not only with the voices of the study participants and their experiences but also with the interpretations and narratives of the original authors, both included and excluded from our analysis. All team members identify as white and cisgender. We recognise how our personal identities and professional experiences informed our engagement with the material, our interpretations, and our collaborative process.

## Results

Of the 4,157 records identified, 28 were critically appraised and summarised in the mixed-method systematic review.

### Critical appraisal

#### Quantitative studies

Of the five studies that used quantitative measurements, one study was classified as having a very high risk of bias,^42^ two studies showed a high risk of bias,^43,44^ one raised some concerns,^45^ and one was assessed as having a low risk of bias (46) (see Supplementary Table 3).

#### Qualitative studies

Using the JBI critical appraisal tool,^38^ we evaluated most studies (n = 15) to have a moderate level of reliability. Additional details regarding the critical assessments are provided in Supplementary Table 4.

### Main description

Of the 28 studies, 23 were qualitative,^46–68^ four were quantitative,^42,43,45,69^ and one was mixed method.^44^ Eleven were conducted in the United States,^2–12^ eight in Australia,^13–16,20–23^ two each in Ireland,^24,25^ and the United Kingdom,^26,27^ and one publication each from Canada,^44^ Germany,^46^ Italy and Spain (Cataluña),^51^ Netherlands,^54^ and Spain.^64^ All studies examined one or more aspects of abortion stigma, according to our theoretical framework (Figure 1). Supplementary Table 5 details the characteristics of all included studies.

**RQ1:** Used definitions for abortion stigma

**RQ2:** Abortion stigma among abortion providers (quantitative studies)

The studies’ origins, sample sizes, measurement tools, and levels of stigma and their associations are shown in Supplementary Table 3. Among the included studies, four utilised the APSS as their primary measurement instrument.^43–45,69^ Notably, one of these studies^43^ also served to evaluate the psychometric properties of the APSS, providing evidence of its validity and reliability within the specific context of the research field. The APSS measures the level of stigma experienced by abortion providers related to their profession on 35 items using a 5-point Likert scale, where a higher sum score indicates more stigma. This includes assessing their sense of being undervalued by society, feelings of shame, perceptions of how others react to their work, and decisions on whether to disclose their involvement in abortion services. The APSS includes a total score and five subscales: Disclosure Management (10 items), Internalised States (10 items), Judgment (7 items), Social Isolation (4 items), and Discrimination (4 items). The five subscales allowed us to provide information on the divergent dimensions of abortion-related stigma. However, only three of the four studies^43,44,69^ reported data for all subscales (see Supplementary Table 7 for a comparison of APSS scores across the four studies).

Across the four studies, total APSS scores (potential range: 35–175) showed measurable levels of abortion provider stigma among providers of HICs. The reported mean scores ranged from 67.8 ^44^ to 76.1,^43^ indicating varying but consistent experiences of stigma. Subscale data also reflected this trend, with higher average scores reported for dimensions such as disclosure management, internalised stigma, and judgment. While some subscales varied slightly across samples, all studies demonstrated that abortion-related stigma persisted among providers in high-income settings, underlining the relevance of stigma as a shared experience despite differing national contexts.

One Australian study used the best–worst scaling method,^42^ a stated preference approach that quantifies the relative importance of items by asking respondents to repeatedly select the most and least important items from subsets of a larger list. This method demonstrated participants’ priorities through systematic trade-offs and provided insight into the sources of stigma that were perceived as the most and least significant. On this scale, general practitioners and registered nurses were asked to rank potential barriers and facilitators of early medical abortion.^42^ Besides anticipated stigma (“Stigma of being known as an early medical abortion provider”), institutional and structural components of stigma (e.g. legal requirements, lack of clinical guidelines, training, information, and supply network), and lack of support by colleagues were rated on the best-worst scaling. Both general practitioners and registered nurses identified several key obstacles in their practice, such as institutional forms of stigma (e.g. absence of established clinical protocols). Additionally, registered nurses highlighted concerns about the potential stigma associated with their involvement in early medical abortion services as a significant barrier.

**RQ3:** Associated factors with abortion stigma (quantitative studies)

All five quantitative studies^42–45,69^ explored abortion-related stigma and its associations with various factors including professional roles, job-related experiences, and psychological outcomes.

#### Professional role and job-related experiences

Stigma varied significantly by professional role, with higher stigma among obstetricians, midwives, and nurses compared to general practitioners.^69^ Counsellors involved in abortion services reported significantly lower stigma scores than nurses.^45^ The study by Haas et al.^28^ clearly showed that nurses, in contrast to general practitioners, perceived that abortion-related stigma was the most impactful barrier to early medical abortion, indicating a tremendous influence of professional roles on the perception of stigma.

Regarding the volume of abortion provision, findings were mixed. Ennis et al.^44^ found that low-volume clinicians reported lower levels of harassment compared with high-volume clinicians. In contrast, Dempsey et al.^69^ found no significant association between abortion provider stigma, measured with the APSS, and the amount of clinical time spent providing abortion care or involvement in emergency care. These findings suggest that the relationship between abortion-related stigma and the extent or type of abortion-related clinical work remains inconclusive.

#### Psychological parameters

Stigma was linked to burnout-related factors across all studies,^2,3,24^ including emotional exhaustion, depersonalisation, and job strain, although the specific subscales and associations varied. In a study by Dempsey et al.,^69^ the APSS subscales of Judgment and Disclosure Management were positively correlated with emotional exhaustion. Conversely, the Internalised States subscale negatively correlated with personal accomplishment. However, the total APSS score was not significantly associated with the Maslach Burnout Inventory.^69^ Janiak et al.^45^ found that higher total APSS scores were associated with increased odds of depersonalisation after controlling for worksite type, job role, and employment status (full-time versus part-time). Martin et al.^43^ showed that APSS scores were positively correlated with the Maslach Burnout Inventory subscales of Emotional Exhaustion and Depersonalisation. Stigma was inversely correlated with the Maslach Burnout Inventory Subscale Personal Accomplishment.

Beyond burnout, stigma was linked to broader occupational stress outcomes. For instance, participants with high stigma had 3.78 times greater odds of experiencing job strain (*p* = 0.03) after controlling for job role and worksite type.^45^ Finally, the total APSS scores were moderately correlated with psychological distress, indicating that stigma may have wider implications for overall mental health beyond workplace-specific burnout.^43^

#### Religion

Religiosity was associated with higher stigma, as providers who regularly attended religious services had higher stigma scores.^45^ However, no other included study has investigated this association.

**RQ4:** Lived experiences concerning abortion stigma (qualitative studies)

The synthesised qualitative results from our meta-aggregation draw upon studies that collectively capture the viewpoints of approximately 996 participants: 917 abortion providers (general practitioners, (obstetricians-)gynaecologists, or doulas), 14 medical students, and 65 clinical staff. We synthesised three main themes: (1) Institutional and structural abortion stigma, (2) Individual abortion stigma, and (3) Mitigating factors. The extracted findings (cf. Supplementary Table 8) were meta-synthesised (cf. Supplementary Table 9), with their corresponding ConQual scores presented in the Supplementary Table 10.

#### Synthesised finding 1: individual abortion stigma

The first finding was synthesised from three categories, comprising 30 extracts from 17 studies,^44,46–52,55–57,60,61,63,65–67^ supported by illustrations (cf. Supplementary Table 8). Individual abortion stigma among healthcare professionals arose from cultural, religious, and “pro-life” narratives leading to harassment, discrimination, and ostracism, which affected their practice, economic well-being, and professional identity.

“*I was really upset when my photos were posted publicly. I can’t be confident I’m safe.*” (p. 1353)^47^

“*Oh, even some of my colleagues … are very set in their views. I found that when they found out that I’m doing these things that they have viewed me differently which is a bit depressing.*” (p. 5)^48^

“*[Y]ou don’t want to put yourself at risk of maybe being targeted by any anti-abortion campaigners if you’re too visible.*” (p. 333)^49^

“*Probably not well … because a lot of doulas are, although some doulas are very open, I think a lot of doulas come from like upper middle-class families that are … you know. They just wouldn’t do that in our area.*” (p. 09)^61^

“*I’m aways away from getting abortion in our practice as a regular treatment option because much of the support staff … I don’t even tell them I do abortions [outside of our primary care practice] because they’re conservative.*” (p. 41)^60^

#### Synthesised finding 2: institutional and structural abortion stigma

##### Institutional abortion stigma

Regarding the workspace, findings from the United States^53^ and the Netherlands^54^ indicated that religious values are embedded within institutional policies, contributing to abortion stigma by restricting access and framing abortion as morally problematic within healthcare settings. Furthermore, the findings of a study in Spain^64^ showed how institutional stigma is reinforced through the exclusion of abortions from mainstream medical training and hospital services. Additionally, two findings from the United States^55,67^ indicated professional isolation and fear of backlash through structural circumstances.

“*When I went to [town], one provider had really been wanting to offer abortion services. … And there had definitely been a demand for them, and … she tried to do it, and … there was a lot of pushback and a lot of picketing.*” (p. 155)^67^

##### Structural abortion stigma

Two findings from Australia^58^ and Spain^64^ emphasised that legal reforms alone are insufficient to reduce stigma or improve access to abortion services. This suggests that structural stigma continued to exist when institutions do not fully integrate abortion into mainstream healthcare. In the United States,^61^ structural stigma was embedded in restrictive laws that create logistical, emotional, and professional barriers to abortion care. Findings from Australia,^48^ Spain,^64^ and the United States^59^ also indicated institutional endorsement of abortion stigma.

“* … there are very few providers that could give any information or even would give any information for fear of retribution or backlash on abortion services, especially here in Georgia.*” (p. 09)^61^

#### Synthesised finding 3: mitigating factors

The third finding was derived from three categories of ten extracts from five studies,^61,62,65,66,68^ supported by illustrations. Factors that may help reduce abortion stigma include social changes and legal reforms that decrease secrecy and stigmatisation, as indicated in Ireland.^65^ Further, findings from Australia^66^ and the United States^62^ indicated that providing judgment-free abortion care might be helpful.^62,66,68^ Finally, practitioners’ dedication to empowering patients through accurate information and championing reproductive rights, was shown by two studies from the United States:^61,68^

“* … there are just too many reasons that abortion care should be accessible. You’re not going to change my mind about that. And I think what really confuses people with me in particular is when I go from saying that abortion care should 10,000% be accessible, and I’m like, oh, yes, but natural birth should also be 10,000% accessible … I can be pro-abortion and also be pro-birth.*” (p. 09–10)^61^

“*It just feels freer, it feels less restrictive and I think it's more respectful of a woman's choice, its empowering a woman to feel that she can freely make a choice that she's free under the legal, you know, umbrella, so it does shift something.*” (p. 28)^65^

### Integration of quantitative and qualitative evidence

#### Supportive evidence

Both quantitative and qualitative studies showed that abortion providers experience not only individual, but also institutional and structural forms of abortion-related stigma.

The quantitative and qualitative findings suggested that abortion providers anticipate stigma that influences disclosure management. This means that they may choose not to disclose abortion care openly as part of their professional practice. Moreover, both study types indicated that abortion providers perceive stigma from different sources, such as their close social environments and colleagues. We also found internalised stigma in both the quantitative and qualitative studies. In qualitative studies, structural stigma was identified, for example, in the form of anti-abortion politics or restricted working conditions, whereas it was only implicitly captured through individual survey items in a single quantitative study.^42^

#### Contradictory evidence

APSS was primarily used to measure abortion-related stigma in quantitative studies. Although it assesses individual and structural stigma, the APSS does not differentiate between them in its subscales, impeding nuanced analysis or comparison with the qualitative findings. In qualitative studies, the authors did not necessarily differentiate between individual, institutional, and structural stigmas in their theoretical framework; however, their findings can be categorised accordingly.

In terms of structural stigma, qualitative studies highlighted anti-abortion policies as impactful catalysts – an important factor that has not been addressed in quantitative studies. However, the prevailing institutional anti-abortion attitudes within the working space must also be considered. Qualitative studies explicitly mentioned perceived stigma of protesters, whereas quantitative studies assessed verbal and physical threats in general.

Moreover, the level of confidence varied between study types. While qualitative studies generally demonstrated a moderate level of confidence, three out of five quantitative studies^42–44^ revealed a (very) high risk of bias and, thus, notable limitations. Consequently, the findings of quantitative studies should be interpreted with caution.

## Discussion

This review significantly expands Hanschmidt et al.'s^6^ findings by incorporating a substantially larger collection of qualitative studies, increasing from two to 24 studies, and offering a more nuanced and context-sensitive understanding of abortion stigma among healthcare professionals in HICs. In contrast, only a few additional quantitative studies were identified (from three to five), and the APSS remains the dominant measure. Therefore, we have not seen much new evidence in the measurement of abortion stigma among abortion providers since the review by Hanschmidt et al.^6^ in 2016.

This review is the first to systematically synthesise this volume of qualitative work in this area, providing updated insights into how abortion provider stigma is shaped by context, profession, and evolving socio-political conditions. By applying the concepts of individual, institutional and structural abortion-related stigma, we were able to illustrate the interconnectedness of broader societal systems, including legal frameworks, cultural norms, healthcare policies, and everyday experiences of abortion providers. Echoing other scholars in the field,^1,4^ we emphasise that these levels of stigma do not operate in isolation; instead, they reinforce each other, shaping institutional practices and contributing to providers’ personal and professional challenges.

At the individual level, our findings illustrate how stigma manifests through abortion providers’ experiences of harassment, discrimination, and social ostracism, often leading to fear, professional caution, and selective disclosure. These patterns were consistent with the findings of Hanschmidt et al.^6^ and support the “legitimacy paradox” theory coined by Harris,^3^ which suggests that abortion stigma discourages providers from discussing their work, contributing to a perception that abortion provision is unusual and non-standard. That noted, Merner et al.^70^ reported in their scoping review that abortion providers, like other health professionals, are motivated by supporting women’s choices and rights as well as by non-judgment, compassion, and altruism, contributing to the normalisation of abortion as routine healthcare. This indicates that although stigma persists, healthcare providers often play a crucial role in confronting stigma and upholding the ethical and professional validity of abortion services. However, as Millar^1^ critically observes, there is a danger of individualising a structural problem.

This is particularly important given that in our findings, individual abortion stigma was closely related to institutional and structural factors. Our review highlights that structural and institutional stigma are indicative of systemic shortcomings by governments in fulfilling their duties under international human rights law to guarantee non-discriminatory, stigma-free access to abortion services.^71^ Cross-country comparisons in our review show that legal and sociocultural contexts shape provider experiences: U.S. providers in conservative states face higher stigma and institutional barriers, while those in liberal regions report less.^43^ Irish providers^69^ experienced notable social isolation despite fewer direct stigma encounters, and Canadian^44^ providers reported the lowest stigma, reflecting a fully decriminalised, supportive healthcare system. This highlights the fact that abortion-related stigma cannot be understood or addressed in isolation from the broader systems that produce and sustain it.^2,4,16^

At the same time, it is entrenched at the institutional level, where abortion services are often marginalised within healthcare systems. These two levels of stigma are closely interconnected, so much so that institutional stigma might persist even when structural barriers are lifted.^58,64^ This highlights the critical need to examine structural and institutional stigma in tandem to fully understand how abortion-related stigma is maintained and reproduced in healthcare settings. This aligns with the research of Millar^1^ and Strong,^4^ who emphasise that abortion-related stigma reflects an unjust system. As noted by Nandagiri et al.,^72^ abortion stigma is one of the many interconnected factors that contribute to structural violence surrounding abortion access and provision, ultimately disrupting sexual and reproductive health. In this light, our findings also contribute to framing abortion-related stigma not only as a social or professional concern but also as a critical sexual and reproductive health and rights issue.

Recognising institutional stigma also means acknowledging that healthcare professionals can both experience^2^ and enact stigma.^6^ However, the studies included in our review primarily focused on the stigma experienced by providers and did not explore how providers themselves participate in the enactment of stigma. This omission may be partially attributable to the nature of the measurement tools used in quantitative studies, which are designed to assess perceived stigma, as well as the framing and prompts employed in qualitative interviews, which often centre on provider experiences rather than their potential role in reproducing stigma. Addressing this gap is essential for future research that aims to comprehensively understand the dynamics of abortion stigma within healthcare systems.

### Strengths and limitations

Our review utilised the JBI mixed-method systematic reviews methodology to analyse quantitative and qualitative data on abortion stigma among providers, offering a comprehensive overview of the current research. Its strengths include a thorough examination of the potential effects and personal experience of abortion stigma. However, the following limitations exist: (1) Our focus on HICs restricts its applicability in other contexts. However, HICs are not homogeneous, and the included studies reflect variations in legal frameworks, healthcare organisation, and sociocultural climates. At the same time, many included settings share broader features, such as professionalised abortion provision, biomedical healthcare systems, and moral discourses around abortion that are often shaped by Christian-influenced norms of sexuality, reproduction, and motherhood. Therefore, some findings may be relevant to other settings with similar legal or service-provision structures. Nevertheless, transferability remains limited, as abortion stigma may be shaped differently in contexts where other religious, cultural, political, or community-based belief systems structure attitudes towards abortion. (2) The meta-aggregation process involves subjective categorisation, which is influenced by individual perspectives. (3) A significant limitation of employing a meta-aggregation method is the tendency to overgeneralise qualitative study results. This additional abstraction level further distorts the aggregated results from the initial context.^73^ (4) Some of the included quantitative studies^42–44^ showed a very high risk of bias and a lack of confidence. Therefore, future research should prioritise high-quality study designs and rigorous methodologies to minimise the risk of bias and enhance the reliability of findings.

## Conclusion

These findings illustrate that abortion stigma is not only an individual phenomenon but also a broader societal one, deeply embedded within healthcare structures and legal systems. Even in countries with liberal abortion laws, stigma persists at institutional and social levels, affecting providers and patients. The integration of quantitative and qualitative evidence reinforces the need for multifaceted interventions, including policy changes, provider protection, and cultural shifts, to reduce abortion stigma and improve healthcare access globally.

Future research should focus on developing nuanced measurement tools to assess stigma across different healthcare contexts and evaluate effective interventions. Efforts must move from the individual level to target institutional and structural drivers. Addressing abortion-related provider stigma is essential to ensuring sustainable access to abortion care and safeguarding the well-being of those who provide it. Efforts to reduce abortion stigma are not only clinically important, but also a necessary component of upholding global commitments to sexual and reproductive rights and justice.

## Supplementary Material

Supplementary Table 3: Included Quantitative Studies

Supplementary Table 7. Comparison of Quantitative studies that used the APSS to assess abortion related stigma among abortion providers

Supplementary Table 1. PRISMA 2020 Checklist

Supplementary Table 5. Main characteristics of the included studies

Supplementary Table 10. Meta-synthesized findings.

Supplementary Table 4 Critical Appraisal of the qualitative studies

Supplementary Table 8. Findings of the included qualitative studies

Supplementary Table 2. Search string

Supplementary Table 6. Abortion stigma definitions

Supplementary Table 9. Synthesized Qualitative Findings

## Footnotes

† In this review, the term abortion provider refers to any individual involved in abortion care, such as practitioners, midwives, nurses, obstetricians, gynecologists, doulas, counsellors, and other staff members working in abortion care.

## Provenance statement

This article was not commissioned and went through external peer review.

## References

[1] Millar E. Abortion stigma as a social process. Womens Stud Int Forum. 2020;78:102328. doi:10.1016/j.wsif.2019.102328

[2] Dempsey B, Callaghan S, Higgins MF. Providers’ experiences with abortion care: a scoping review. PLoS One. 2024;19:e0303601. doi:10.1371/journal.pone.0303601 38950040 PMC11216598

[3] Harris LH, Martin L, Debbink M, et al. Physicians, abortion provision and the legitimacy paradox. Contraception. 2013;87(1):11–16. doi:10.1016/j.contraception.2012.08.031 23063339

[4] Strong J, Coast E, Nandagiri R. Abortion, stigma, and intersectionality. In: P. Liamputtong, editor. Handbook of social sciences and global public health [Internet]. Cham: Springer International Publishing; 2023. p. 1–22. Available from: https://link.springer.com/10.1007978-3-030-96778-9_103-1

[5] Sorhaindo AM, Lavelanet AF. Why does abortion stigma matter? A scoping review and hybrid analysis of qualitative evidence illustrating the role of stigma in the quality of abortion care. Soc Sci Med. 2022;311:115271. doi:10.1016/j.socscimed.2022.115271 36152401 PMC9577010

[6] Hanschmidt F, Linde K, Hilbert A, et al. Abortion stigma: a systematic review. Perspect Sex Reprod Health. 2016;48(4):169–177. doi:10.1363/48e8516 27037848

[7] Martin LA, Debbink M, Hassinger J, et al. Measuring stigma among abortion providers: assessing the abortion provider stigma survey instrument. Women Health. 2014;54(7):641–661. doi:10.1080/03630242.2014.919981 25061823

[8] Martin LA, Debbink M, Hassinger J, et al. Abortion providers, stigma and professional quality of life. Contraception. 2014;90(6):581–587. doi:10.1016/j.contraception.2014.07.011 25131444

[9] O’Donnell J, Weitz TA, Freedman LR. Resistance and vulnerability to stigmatization in abortion work. Soc Sci Med. 2011;73(9):1357–1364. doi:10.1016/j.socscimed.2011.08.019 21940082

[10] Harris LH, Debbink M, Martin L, et al. Dynamics of stigma in abortion work: findings from a pilot study of the providers share workshop. Soc Sci Med. 2011;73(7):1062–1070. doi:10.1016/j.socscimed.2011.07.004 21856055

[11] Norris A, Bessett D, Steinberg JR, et al. Abortion stigma: a reconceptualization of constituents, causes, and consequences. Womens Health Issues. 2011;21(3):S49–S54. doi:10.1016/j.whi.2011.02.010 21530840

[12] Hughes EC. Mistakes at work. Can J Econ Polit Sci. 1951;17(3):320–327. doi:10.2307/137687

[13] Joffe C. What abortion counselors want from their clients. Soc Probl. 1978;26(1):112–121. doi:10.2307/800436

[14] Kumar A, Hessini L, Mitchell EMH. Conceptualising abortion stigma. Cult Health Sex. 2009;11(6):625–639. doi:10.1080/13691050902842741 19437175

[15] Goffman E. Stigma: notes on the management of spoiled identity. New York: Simon & Schuster; 1963.

[16] Footman K. Structural stigmatisation of abortion in the health system: perspectives of abortion care-seekers, providers, managers, and funders in England and Wales. Soc Sci Med. 2025;365:117566. doi:10.1016/j.socscimed.2024.117566 39631301

[17] Pescosolido BA, Martin JK. The stigma complex. Annu Rev Sociol. 2015;41:87–116. doi:10.1146/annurev-soc-071312-145702 26855471 PMC4737963

[18] Dean LT, Thorpe RJ. What structural racism is (or is not) and how to measure it: clarity for public health and medical researchers. Am J Epidemiol. 2022;191(9):1521–1526. doi:10.1093/aje/kwac112 35792088 PMC9437815

[19] Hatzenbuehler ML, Link BG. Introduction to the special issue on structural stigma and health. Soc Sci Med. 2014;103:1–6. doi:10.1016/j.socscimed.2013.12.017 24445152

[20] Rai SS, Syurina EV, Peters RMH, et al. Non-communicable diseases-related stigma: a mixed-methods systematic review. Int J Environ Res Public Health. 2020;17(18):6657. doi:10.3390/ijerph17186657 32932667 PMC7559120

[21] Shellenberg KM, Tsui AO. Correlates of perceived and internalized stigma among abortion patients in the USA: an exploration by race and Hispanic ethnicity. Int J Gynaecol Obstet. 2012;118(S2):S152–S159. doi:10.1016/S0020-7292(12)60015-0 22920620

[22] Cockrill K, Nack A. “I’m not that type of person”: managing the stigma of having an abortion. Deviant Behav. 2013;34(12):973–990. doi:10.1080/01639625.2013.800423

[23] Link BG, Phelan JC. Conceptualizing stigma. Annu Rev Sociol. 2001;27(1):363–385. doi:10.1146/annurev.soc.27.1.363

[24] Tyler I, Slater T. Rethinking the sociology of stigma. Sociol Rev. 2018;66(4):721–743. doi:10.1177/0038026118777425

[25] Tyler I. Resituating Erving Goffman: from stigma power to black power. Sociol Rev. 2018;66(4):744–765. doi:10.1177/0038026118777450

[26] Link BG, Phelan J. Stigma power. Soc Sci Med. 2014;103:24–32. doi:10.1016/j.socscimed.2013.07.035 24507908 PMC4451051

[27] Herek GM. Sexual stigma and sexual prejudice in the United States: a conceptual framework. In: Hope DA, editor. Contemporary perspectives on lesbian, gay, and bisexual identities. New York: Springer; 2009. p. 65–111. Available from: http://link.springer.com/10.1007978-0-387-09556-1_4 10.1007/978-0-387-09556-1_4 19230525

[28] Górska P, Bilewicz M, Winiewski M. Invisible to the state. institutional sexual stigma and collective action of LGB individuals in five East European countries. Group Process Intergroup Relat. 2017;20(3):367–381. doi:10.1177/1368430216684646

[29] Human Rights Watch. Reproductive rigths and abortion [Internet]. 2023. Available from: https://www.hrw.org/topic/womens-rights/reproductive-rights-and-abortion

[30] Bernard M, Niemann J, Weinhold L, et al. Abortion stigma among abortion seekers, healthcare professionals and the public in high-income countries: a mixed-methods systematic review protocol. BMJ Open. 2024;14(1):e076602. doi:10.1136/bmjopen-2023-076602 PMC10806672 38238049

[31] Singh S, Remez L, Sedgh G, et al. Abortion Worldwide 2017: uneven progress and unequal access [Internet]. 2018 [cited 2026 Jan 13]. Available from: https://www.guttmacher.org/sites/default/files/report_pdf/abortion-worldwide-2017.pdf

[32] Bearak J, Popinchalk A, Ganatra B, et al. Unintended pregnancy and abortion by income, region, and the legal status of abortion: estimates from a comprehensive model for 1990–2019. Lancet Glob Health. 2020;8(9):e1152–e1161. doi:10.1016/S2214-109X(20)30315-6 32710833

[33] Page MJ, Moher D, Bossuyt PM, et al. PRISMA 2020 explanation and elaboration: updated guidance and exemplars for reporting systematic reviews. BMJ [Internet]. 2021;372:n160. Available from: https://www.bmj.com/content/372/bmj.n160 10.1136/bmj.n160 PMC8005925 33781993

[34] Stern C, Lizarondo L, Carrier J, et al. Methodological guidance for the conduct of mixed methods systematic reviews. JBI Evidence Synth. 2020;18(10):2108–2118. doi:10.11124/JBISRIR-D-19-00169 32813460

[35] The World Bank. 2023. World Bank country and lending groups. Available from: https://datahelpdesk.worldbank.org/knowledgebase/articles/906519-world-bank-country-and-lending-groups#:~:text=

[36] Ouzzani M, Hammady H, Fedorowicz Z, et al. Rayyan—a web and mobile app for systematic reviews. Syst Rev. 2016;5(1):210. doi:10.1186/s13643-016-0384-4 27919275 PMC5139140

[37] ROBINS-E Deveolpment Group. Risk of Bias in Non-randomized Studies - of Exposure (ROBINS-E) [Internet]. Available from: https://www.riskofbias.info/welcome/robins-e-tool

[38] Lockwood C, Munn Z, Porritt K. Qualitative research synthesis: methodological guidance for systematic reviewers utilizing meta-aggregation. Int J Evid Based Healthc. 2015;13(3):179–187. doi:10.1097/XEB.0000000000000062 26262565

[39] Krippendorff K. Content analysis: an introduction to its methodology [internet]. Thousand Oaks (CA): SAGE Publications, Inc.; 2019; Available from: https://methods.sagepub.com/book/content-analysis-4e

[40] Lockwood C, Porritt K, Munn Z, et al. Systematic reviews of qualitative evidence. In: Aromataris E, Lockwood C, Porritt K, et al., editors. JBI Manual for evidence synthesis. 2024. doi:10.46658/JBIMES-24-02

[41] Munn Z, Porritt K, Lockwood C, et al. Establishing confidence in the output of qualitative research synthesis: the ConQual approach. BMC Med Res Methodol. 2014;14(1):108. doi:10.1186/1471-2288-14-108 25927294 PMC4190351

[42] Haas M, Church J, Street DJ, et al. How can we encourage the provision of early medical abortion in primary care? Results of a best–worst scaling survey. Aust J Prim Health. 2023;29(3):252–259. doi:10.1071/PY22130 36473159

[43] Martin LA, Hassinger JA, Seewald M, et al. Evaluation of abortion stigma in the workforce: development of the revised abortion providers stigma scale. Womens Health Issues. 2018;28(1):59–67. doi:10.1016/j.whi.2017.10.004 29133064

[44] Ennis M, Renner RM, Olure B, et al. Experience of stigma and harassment among respondents to the 2019 Canadian abortion provider survey. Contraception. 2023;124:110083. doi:10.1016/j.contraception.2023.110083 37263373

[45] Janiak E, Freeman S, Maurer R, et al. Relationship of job role and clinic type to perceived stigma and occupational stress among abortion workers. Contraception. 2018;98(6):517–521. doi:10.1016/j.contraception.2018.07.003 30053400

[46] Baier A, Behnke AL. Barriers to abortion provision: a qualitative study among medical students and gynecologists in Berlin, Germany. Contraception. 2024;130:110325. doi:10.1016/j.contraception.2023.110325 37935352

[47] Chowdhary P, Newton-Levinson A, Rochat R. “No one does this for the money or lifestyle”: abortion providers’ perspectives on factors affecting workforce recruitment and retention in the southern United States. Matern Child Health J. 2022;26(6):1350–1357. doi:10.1007/s10995-021-03338-6 34997437 PMC9132807

[48] Dawson AJ, Nicolls R, Bateson D, et al. Medical termination of pregnancy in general practice in Australia: a descriptive-interpretive qualitative study. Reprod Health. 2017;14(1):39. doi:10.1186/s12978-017-0303-8 28288649 PMC5348908

[49] Deb S, Subasinghe AK, Mazza D. Providing medical abortion in general practice: general practitioner insights and tips for future providers. Aust J Gen Pract. 2020;49(6):331–337. doi:10.31128/AJGP-01-20-5198 32464728

[50] de Moel-Mandel C, Taket A, Graham M. Identifying barriers and facilitators of full service nurse-led early medication abortion provision: qualitative findings from a Delphi study. Aust J Adv Nurs. 2021;38(1):18–26. doi:10.37464/2020.381.144

[51] De Zordo S. From women’s ‘irresponsibility’ to foetal ‘patienthood’: obstetricians-gynaecologists’ perspectives on abortion and its stigmatisation in Italy and Cataluña. Glob Public Health. 2018;13(6):711–723. doi:10.1080/17441692.2017.1293707 28278744

[52] Fay V, Thomas S, Slade P. Maternal–fetal medicine specialists’ experiences of conducting feticide as part of termination of pregnancy: a qualitative study. Prenat Diagn. 2016;36(1):92–99. doi:10.1002/pd.4720 26531671

[53] Hasselbacher LA, Hebert LE, Liu Y, et al. “My hands are tied”: abortion restrictions and providers’ experiences in religious and nonreligious health care systems. Perspect Sex Reprod Health. 2020;52(2):107–115. doi:10.1363/psrh.12148 32597555

[54] Holten L, de Goeij E, Kleiverda G. Permeability of abortion care in The Netherlands: a qualitative analysis of women’s experiences, health professional perspectives, and the internet resource of women on web. Sex Reprod Health Matters. 2021;29(1):162–179. doi:10.1080/26410397.2021.1917042 PMC8118432 33975533

[55] Homaifar N, Freedman L, French V. “She’s on her own”: a thematic analysis of clinicians’ comments on abortion referral. Contraception. 2017;95(5):470–476. doi:10.1016/j.contraception.2017.01.007 28131650

[56] Hulme-Chambers A, Clune S, Tomnay J. Medical termination of pregnancy service delivery in the context of decentralization: social and structural influences. Int J Equity Health. 2018;17(1):172. doi:10.1186/s12939-018-0888-8 30463561 PMC6249871

[57] Kavanagh A, Wielding S, Cochrane R, et al. ‘Abortion’ or ‘termination of pregnancy’? Views from abortion care providers in Scotland, UK. BMJ Sex Reprod Health. 2018;44(2):122–127. doi:10.1136/bmjsrh-2017-101925 29921635

[58] Keogh LA, Newton D, Bayly C, et al. Intended and unintended consequences of abortion law reform: perspectives of abortion experts in Victoria, Australia. J Fam Plann Reprod Health Care. 2017;43(1):18–24. doi:10.1136/jfprhc-2016-101541 27913574

[59] Kim E, Singh S, Bommaraju A, et al. “We have to respect that option”: the abortion aversion complex in safety-net healthcare organizations. Soc Sci Med. 2021;291:114468. doi:10.1016/j.socscimed.2021.114468 34757239

[60] Lee CM, Johns SL, Stulberg DB, et al. Barriers to abortion provision in primary care in New England, 2019–2020: a qualitative study. Contraception. 2023;117:39–44. doi:10.1016/j.contraception.2022.08.001 35970423

[61] Lindsey A, Narasimhan S, Sayyad A, et al. “I can be pro-abortion and pro-birth”: Opportunities and challenges for full spectrum care among doulas in Georgia. Front Glob Womens Health 2023;4:966208. doi:10.3389/fgwh.2023.966208 PMC10014539 36937040

[62] Mainey L, O’Mullan C, Reid-Searl K. Unfit for purpose: a situational analysis of abortion care and gender-based violence. Collegian. 2022;29(5):557–565. doi:10.1016/j.colegn.2022.01.003

[63] McLeod C, Pivarnik K, Flink-Bochacki R. Individual abortion providers’ experiences with targeted harassment in the United States. Contraception. 2022;107:42–47. doi:10.1016/j.contraception.2021.09.014 34728183

[64] Rostagnol S. Abortion in Andalusia: women’s rights after the Gallardón Bill. Antropologia. 2018;5(2):113–136. doi:10.14672/ada20181460113-136

[65] Ryan M, Nolan A, Vallières F. Lifting the cloak of secrecy: experiences of providing crisis pregnancy counselling in a changing legislative context in Ireland. Couns Psychother Res. 2022;22(1):22–31. doi:10.1002/capr.12476

[66] Singh R, Mazza D, Moloney L, et al. General practitioner experiences in delivering early medical abortion services to women from culturally and linguistically diverse backgrounds. Aust J Gen Pract. 2023;52(8):557–564. doi:10.31128/AJGP-07-22-6485 37532441

[67] Summit AK, Lague I, Dettmann M, et al. Barriers to and enablers of abortion provision for family physicians trained in abortion during residency. Perspect Sex Reprod Health. 2020;52(3):151–159. doi:10.1363/psrh.12154 33051986

[68] Warren E, Kissling A, Norris AH, et al. “I felt like I was a bad person … which I’m not”: stigmatization in crisis pregnancy centers. SSM Qual Res Health. 2022;2:100059. doi:10.1016/j.ssmqr.2022.100059

[69] Dempsey B, Favier M, Mullally A, et al. Exploring providers’ experience of stigma following the introduction of more liberal abortion care in the Republic of Ireland. Contraception. 2021;104(4):414–419. doi:10.1016/j.contraception.2021.04.007 33864811

[70] Merner B, Haining CM, Willmott L, et al. Health providers' reasons for participating in abortion care: A scoping review. Women's Health. 2024;20. doi:10.1177/17455057241233124 PMC10908244 38426387

[71] Human Rights Watch. Q&A: access to abortion is a human right. Human Rights Watch [Internet]. 2022 [cited 2026 Jan 13]. Available from: https://www.hrw.org/news/2022/06/24/qa-access-abortion-human-right

[72] Nandagiri N, Coast E, Strong J. COVID-19 and abortion: making structural violence visible. Int Perspect Sex Reprod Health. 2020;46(Supplement 1):83. doi:10.1363/46e1320 33326403

[73] Bergdah E. Is meta-synthesis turning rich descriptions into thin reductions? A criticism of meta-aggregation as a form of qualitative synthesis. Nurs Inq. 2019;26:e12273. 10.1111/nin.12273 30667158

