# Correction

**DOI:** 10.1080/26410397.2025.2695541

**Published:** 2026-07-06

**Article title:** Abortion stigma among abortion providers in high-income countries: a mixed methods systematic review

**Authors:** Marie Bernard, Jana Niemann, Dennis Jepsen, Nadja Freymüller, Laura Weinhold, Céline Miani, Claudia Luck-Sikorski

**Journal:** *Sexual and Reproductive Health Matters*

**Bibliometrics:** Volume 33, Number 1, pages 1-17

**DOI:** http://dx.doi.org/10.1080/26410397.2026.2668884

When the article was received, the Results section was missing the text for Research Question 1. The missing text has now been added.

**RQ1: Used definitions for abortion stigma**

Six studies^43,45,56,59,64,65^ referred to Kumar, Hessini, and Mitchell’s^14^ definition of abortion stigma, four studies^43,59,64,69^ utilized Norris et al.’s^11^ definition of abortion stigma, another four studies^45,52,63,69^ referred to the definition by Harris et al.^3^ and three studies^45,65,69^ referred to O’Donnell et al.^9^ One study^68^ exclusively used a generic definition of stigma, whereas the vast majority of 18 studies^42,44,46-51,53–55,57,58,60–62,66,67^ did not explicitly state any definition (cf. Supplementary Table 6).

