# Supplementary material for: Abortion stigma among abortion providers in high-income countries: a mixed methods systematic review
Source: Sex Reprod Health Matters. 2026 May 22;33(1):2668884. doi: 10.1080/26410397.2026.2668884 (PMC13276811; doi:10.1080/26410397.2026.2668884)
Supplement: Supplementary Table 3: Included Quantitative Studies [file ZRHM_A_2668884_SM5963.docx]

Supplementary Table 3: Included Quantitative Studies

| Study | Measurement tool | Prevalence (%)/ Level of Stigma (Mean, SD) | Explored Associations with correlations |
| --- | --- | --- | --- |
| Martin et al. (2018)^3^  Country: USA (nationwide)  Sample n=315 | **Abortion Provider Stigma Scale (APSS)**, α =0.924  5 subscales:   - disclosure management - internalized states - judgment - social isolation - discrimination | **Total APSS score:** M=76.1 (*SD* =17.4)  Subscales:   - Disclosure management: M=24.0 *SD*=8.5) - Internalized states: M= 22.0 (*SD* =4.2) - Judgment: M=15.1 (*SD*=4.8) - Social isolation: M= 9.4 (*SD*=3.6) - Discrimination: M= 5.4 (*SD*=1.9) | - APSS was correlated with psychological distress (r=0.40; p<.001) - APSS was correlated with Maslach Burnout Inventory subscales *emotional exhaustion* (r=0.27; p<.001), and *depersonalization* (r=0.23; p<.001) - APSS was inversely correlated with Maslach Burnout Inventory subscale *personal accomplishment* (r=0.15; p<.05) |
| Dempsey et al. (2021)^1^  Country: IRE  Sample n=156 | **Abortion Provider Stigma Scale (APSS)**  5 subscales:   - disclosure management - internalized states - judgment - social isolation - discrimination | **Total APSS score:** M=70.9 (*SD*=15.35)  Subscales:   - Disclosure management M=21.43 (*SD*=6.74) - Internalized states M=21.91 (*SD*=5.31) - Judgment M=13.44 (*SD*=4.09) - Social isolation M=10.36 (*SD*=3.47) - Discrimination Median=4 (IQR=4,4) | - Obstetricians (b=10.51, 95% CI 3.16–17.86) and midwives/nurses (b=10.88, 95% CI 2.3–19.47) reported higher stigma than General Practitioners - APSS was not associated with gender, region, proportion of clinical time, and involvement in emergency care - APSS total score was not associated with Maslach Burnout Inventory - APSS subscales *judgement* & *disclosure management* are positively correlated with emotional exhaustion - APSS subscale *internalized states* is negatively correlated with personal accomplishment |
| Ennis et al. (2023)^3^  Country: CAN  Sample n=354 | **Abortion Provider Stigma Scale (APSS)**  5 subscales:   - disclosure management - internalized states - judgment - social isolation - discrimination   Harassment | **Total APSS score:** M=67.8 (*SD*=17.2)  Subscales:   - Disclosure management: M=22.5 (*SD*=8.6) - Internalized states: M=18.6 (*SD*=4.9) - Judgment: M=12.8 (*SD*=4.4) - Social isolation: M=9.0 (*SD*=3.6) - Discrimination: M=4.6 (*SD*=1.4)   *Reported harassment among clinicians:*   - Low-volume clinicians: 8% (n=14) - High-volume clinicians: 21% (n=25) - 18% of all clinicians seriously considered changing the care they provide due to harassment   *Reported types of harassment among clinicians:*   - Picketing at work: 70% (n=28) - Picketing at home: 13% (n=5) - Vandalism: <5 affected - Denied positions at hospitals: <5 affected   *Reported Harassment among administrators:*   - 47% (n=18) of administrators reported harassment at their facility in 2019 - Most common type of harassment: Picketing without blocking or contact (83%, n=15) - Median number of harassment events per facility in 2019: 6.2 (IQR: 2.0–106.3) | - Few reports of harassment among low-volume clinicians - More reports of harassment among high-volume clinicians - Highest rate of reporting harassment among administrators |
| Janiak et al. (2018)^2^  Country: USA  (Massachusetts)  Sample n=136 | **Abortion Provider Stigma Scale (APSS)** | **Total APSS score:** M=69 (*SD*=15.2) | - Higher APSS score among participants who regularly attend religious services (b=9.5; p=0.03) - Lower APSS score among counselors compared to nurses (b=-11.0; p ≤0.001) - Job Strain: Workers with high stigma had 3.78 (p=0.03) times higher odds of experiencing job strain (accounting for job role and worksite type) - Burnout: increase in total APSS score was associated with higher odds of experiencing Depersonalization (accounting for worksite type, job role and full- vs. part-time work) |
| Haas et al. (2022)^4^  Country: AUS  Sample n=300 | Best-worst Scaling Barriers to the provision of Early medical abortion (EMA); 15 potential barriers | - Stigma is the most important barrier to the provision of EMA for registered nurses - Stigma is the 12th important barrier to the provision of EMA for general practitioners | - Stigma of being known as an EMA provider most important barrier among registered nurses - Structural barriers (legal requirements, lack of clinical guidelines, lack of support) are important barriers |

NOTE: 1 ROBINS-Score (Risk of Bias): ^1^=low risk; ^2^=some concerns; ^3^=high risk; ^4^=very high risk; APSS= Abortion Provider Stigma Scale; MBI=Maslach Burnout Inventory
