# Supplementary material for: Abortion stigma among abortion providers in high-income countries: a mixed methods systematic review
Source: Sex Reprod Health Matters. 2026 May 22;33(1):2668884. doi: 10.1080/26410397.2026.2668884 (PMC13276811; doi:10.1080/26410397.2026.2668884)
Supplement: Supplementary Table 7. Comparison of Quantitative studies that used the APSS to assess abortion related stigma among abortion providers [file ZRHM_A_2668884_SM5962.docx]

Supplementary Table 7. Comparison of Quantitative studies that used the APSS to assess abortion related stigma among abortion providers

| Study | Martin 2018 [24] | Dempsey 2021 [38] | Ennis 2023 [36] | Janiak 2018 [37] |
| --- | --- | --- | --- | --- |
| n | N=315 | N=156 | N=354 | N=136 |
| Score range | 39-140 | 37-134 | NA | 42-118 |
| Total score | M=76.1 (SD=17.4) | M=70.9 (SD=15.35) | M=67.8 (SD=17.2) | M=69  (SD=15.2) |
| Disclosure  Management | M=24.0 (SD=8.5) | M=21.43 (SD=6.74) | M=22.5 (SD=8.6) | NA |
| Internalized States | M= 22.0 (SD=4.2) | M=21.91 (SD=5.31) | M=18.6 (SD=4.9) | NA |
| Judgment | M=15.1 (SD=4.8) | M=13.44 (SD=4.09) | M=12.8 (SD=4.4) | NA |
| Social  Isolation | M= 9.4  (SD=3.6) | M=10.36 (SD=3.47) | M=9.0  (SD=3.6) | NA |
| Discrimination | M= 5.4  (SD=1.9) | Median=4  (IQR=4.4) | M=4.6  (SD=1.4) | NA |

Note: APSS=Abortion Provider Stigma Scales: sum score (possible range of total score: 35-175); IQR=interquartile range
