# Supplementary material for: Abortion stigma among abortion providers in high-income countries: a mixed methods systematic review
Source: Sex Reprod Health Matters. 2026 May 22;33(1):2668884. doi: 10.1080/26410397.2026.2668884 (PMC13276811; doi:10.1080/26410397.2026.2668884)
Supplement: Supplementary Table 5. Main characteristics of the included studies [file ZRHM_A_2668884_SM5960.docx]

Supplementary Table 5. Main characteristics of the included studies

| **Study &**  **Country** | | **Participant characteristics and sample size** | **Goal of the study/ Phenomena of interest** | **Methodology** | **Methods for data collection and analysis** (QUAL only) | **Description of main results** |
| --- | --- | --- | --- | --- | --- | --- |
| **Quantitative studies** | | | | | | |
| Dempsey et al. (2021) | Ireland | Sample size: n=156  Profession: 66.7% GP; 18% obstetrician/gynecologists; 10.9% midwifes; 3.8% nurses/service administrators/anesthesiologists  Age:  20s: 3.2%  30s: 27.6%  40s: 42.3%  50s: 19.6%  60s: 7.1%  70s: 0.3% | The study sought to investigate healthcare professionals' encounters with abortion-related stigma in Ireland. It also compared stigma levels experienced by Irish abortion providers to previously reported data and examined potential demographic trends within the participant group. Furthermore, the investigation explored the relationship between stigma and burnout among medical personnel involved in abortion care. | Quantitative approach | **/** | The study revealed that Irish abortion care providers experienced lower overall stigma compared to their counterparts in the USA, with reduced levels of judgment and discrimination but higher levels of social isolation. Differences in stigma were observed based on job roles, with obstetricians/gynecologists and midwives reporting higher scores than general practitioners. Stigma was significantly associated with burnout, particularly through judgment and social isolation. Additionally, Irish providers faced fewer instances of harassment and violence, likely due to stronger public support following the legalization of abortion in Ireland. |
| Haas et al. (2022) | Australia | Sample size: 300  Profession: 50% General Practitioner; 50& registered nurses Age:  20–29 years: 1.3% (GPs), 6.0% (RNs)  30–39 years: 23.3% (GPs), 36.7% (RNs)  40–49 years: 32.0% (GPs), 27.3% (RNs)  50–59 years: 23.3% (GPs), 18.0% (RNs)  60–69 years: 18.0% (GPs), 12.0% (RNs)  70–79 years: 2.0% (GPs), 0.0% (RNs) | The study identified that the most critical facilitators for the provision of EMA in primary care was the availability of a community of practice. The most significant barriers included the stigma associated with being known as an EMA provider (particularly for nurses), a lack of clinical guidelines, the substantial time and effort required for information provision and counseling, and challenges related to patient follow-up. | Quantitative approach | **/** | The study aimed to quantitatively assess the relative importance of barriers and facilitators to the provision of early medical abortion (EMA) in primary care. Using a best–worst scaling (BWS) survey, it explored the perspectives of general practitioners (GPs) and registered nurses (RNs) in Australia to identify the key factors that motivate and support EMA provision and those that hinder it, ensuring primary care structures effectively address these issues. |
| Janiak et al. (2018) | United states, Massachusetts | Sample size: n=136  Profession: 50% nurses, 41.2% medical assistants (MAs), and 8.8% counselors Age: M 39.2 (SD=14.0) | The study found that abortion workers with higher stigma scores experienced significantly higher levels of job strain and burnout, particularly emotional exhaustion and depersonalization. Counselors had lower stigma levels than nurses but reported higher emotional strain and lower professional accomplishment. Nearly half of participants reported moderate (35%) or high (12%) levels of emotional exhaustion. Workers exposed to stigma were 3.78 times more likely to report job strain, emphasizing the impact of stigma on occupational stress and mental health. | Quantitative approach | **/** | The study aimed to quantitatively assess the relationship between stigma and occupational stress among clinical abortion workers. This study sought to address gaps by exploring associations between worksite type, worker roles, and occupational psychological health. |
| Martin et al. (2018) | United states | Sample size: 315  Profession: 30% medical assistants, 23% managers, 14% nurses, 11% physicians, 9% advanced care providers, 7% counselors, 5% participants with multiple job types, and 1% senior leadership Age:  18–24: 6%  25–34: 38%  35–44: 30%  45–54: 13%  55–64: 10%  > 65: 3% | The study developed a revised version of the Abortion Providers Stigma Scale (APSS), which evaluates five dimensions of stigma: disclosure management, internalized stigma, social isolation, judgment, and discrimination. The revised scale proved to be psychometrically valid and reliable. Findings showed that while most providers felt pride in their work, many still experienced stigma, particularly internalized stigma and social isolation. | Quantitative approach | **/** | The study aimed to revise and evaluate the psychometric properties of the Abortion Providers Stigma Scale (APSS) to better capture abortion providers’ experiences of stigma. Building on the original 13-item version, which focused on disclosure management, resilience, and discrimination, the study sought to address its limitations by incorporating additional domains, such as internalized stigma, and revising item wording for clarity. |
| Ennis et al. (2023) | Canada | Sample size: n=354  Profession: 92.7% Physicians and nurses (clinicians) who provided abortion care; 11% abortion service administrators Age: NA | Among low-volume clinicians, 14% reported harassment compared to 2% of high-volume clinicians. Common forms included picketing (28 cases) and vandalism (fewer than five cases). Forty-seven percent of clinicians worked in legislated safe access zones (“bubble zones”), which 24% of administrators viewed as beneficial for reducing harassment. The mean stigma score on the APSS was 67.8 out of 175. Harassment was notably more frequent for high-volume providers. | Mixed Methods approach |  | The researchers conducted an investigation to examine the experiences of stigma and harassment faced by abortion providers and administrators in Canada. |

**Supplementary Table 2 (continued)**

| **Study &**  **Country** | | **Participant characteristics and sample size** | **Goal of the study/ Phenomena of interest** | **Methodology** | **Methods for data collection and analysis** (QUAL only) | **Description of main results** |
| --- | --- | --- | --- | --- | --- | --- |
| **Qualitative studies** | | | | | | |
| Baier & Behnke (2024) | Germany | 14 medical students (first to sixth semester) and 4 gynecologists (gyns). Age: Gyns: 37.5 (27–46); MS: 25.2 (18–41)  Gender: Gyns: Female (n=4), MS: Female (n=11), male (n=3) Religion: Gyns: Christian (n=3), No answer (n=1); MS: Christian (n=8), No answer (n=6) | The authors sought to explore the attitudes of medical students and obstetrician-gynecologists toward various aspects of abortion and to identify barriers that might prevent them from providing abortion care. | Descriptive qualitative | The researchers conducted a qualitative content analysis of the interview data. They audio-recorded and transcribed the interviews verbatim, anonymized personal information, and used MAXQDA software to organize and analyze the data. The researchers derived their coding system based on principles of qualitative content analysis, creating categories in a deductive-inductive manner. They focused the analysis on barriers to abortion care, synthesizing interview content by questionnaire topic and identifying relevant themes. During the interview phase, the researchers determined thematic saturation for medical students, but were unable to reach saturation for physicians due to recruitment challenges. | The study found that abortion is widely perceived as a taboo and stigmatized procedure, both in society in general and within the medical profession in particular. Many interviewees feared stigmatization and hostility from their professional and personal environments when discussing or providing abortion. The study suggests that the regulation of abortion in the German Criminal Code is likely to perpetuate this abortion-related stigma experienced by health care providers. |
| Chowdhary et al. (2022) | United States, Alabama, Florida, Georgia, Louisiana, Mississippi, North Carolina, and Texas. | 11 Physicians who provided abortion services Sex: female (n=7), male (n=5) Age: <50 (n=8)  Race: Individuals of colour (n=7) | The paper examines abortion providers' perspectives on the personal and professional challenges of practicing in the region. The study aims to identify potential opportunities to mitigate these problems. | Descriptive qualitative | The researchers conducted in-depth interviews and used grounded theory methodology to code and group the data. They refined their codebook and identified four overarching themes. They presented their findings for validation and continued to refine their conceptual framework through group discussion and exploration of connections between codes. | Abortion providers face challenges such as restrictive laws, institutional segregation, lack of training, safety concerns, identity struggles, and marginalization within their profession. They experience stigma and isolation, particularly in the South, and struggle to maintain professional privacy. Marginalization within the medical community limits their practice, financial gain, and development. Providers also face safety concerns and engage in protective behaviors. They struggle with their identity and sense of belonging, which are affected by their professional disclosure. |
| Dawson et al. (2017) | Australia, NSW | 32 GPs (8 MTOP providers, 24 non MTOP providers)  focus group (N = 4)  Gender: Female (n=24, 75%), male (n=9, 28.1%) Role: General practitioner (n=31, 96.8%), GP/surgeon (n=1, 3.1%) Provision of MTOP: Provider (n=8, 25%) Non-provider (n=24, 75%) | The study aimed to describe the pathways for women seeking abortion through general practice, the factors that determine these pathways from the perspective of general practitioners, and related workforce issues. | Descriptive-interpretive qualitative research study | Semi-structured interviews and a focus group were conducted with family physicians. Data were analyzed using a thematic analysis approach. Data were coded by multiple experts and emergent themes were identified through group discussion. Patterns and discrepant themes were explored across the data. | The study found that while some family physicians were interested in providing medical abortion, they were concerned about the stigma associated with it and the impact it might have on their practice and relationships with colleagues. GPs who provided MTOP tended to be women who were committed to comprehensive sexual and reproductive health care, but often faced disapproval and judgmental attitudes from colleagues, friends and family. MTOP providers were generally reluctant to promote the service, fearing a backlash from anti-abortion activists. Some general practitioners did not perceive any stigma associated with providing abortion, while others were discouraged from providing MTOP because of personal beliefs or the stigma of being labeled an “abortionist. |
| Deb et al. (2020) | Australia | 25 General Practitioners Sex: Female (n=18), male (n=7) Age: 30–39 (n=13), 40–49 (n=9), 50–59 (n=2), ≥60 (n=1)  Years providing medical abortion: <1 year (n=6), ≥1 but <2 years (n=6), ≥2 but <3 years (n=3), ≥3 but <4 years (n=5), ≥4 but <5 years (n=3), ≥5 years (n=2) | The ​​aim of this study was to describe GP medical abortion ​​delivery models. | Qualitative descriptive approach | The study used semi-structured telephone interviews with general practitioners (GPs) providing medical abortion services across the country. Participants were recruited using purposive and snowball sampling, and demographic information was collected. The interview guide was based on a quality framework and included topics such as establishment of provision, structure of the medical abortion model, involvement of other clinicians, acceptability, improvements and advice. Interviews were transcribed verbatim, coded using thematic analysis by two researchers, and findings interpreted using the quality framework. | In summary, the paper shows that abortion stigma is a significant barrier faced by GPs providing medical abortion services in Australia, leading some to operate discreetly and avoid openly advertising their services. Many participants who had announced their decision to begin providing medical abortion services in their practice simultaneously restricted promotion of the service due to fear and stigma. |
| Moel-Mandel (2021) | Australia, Victoria | 24 panellists, nurses (n=10), physicians (n=7), professionals belonging to the ‘other’ group (n=7). | The study explores the views of Delphi panelists on the factors that can influence the implementation of this model. | Descriptive qualitative, Capability, Opportunity and Motivation-Behaviour (COM-B). | The study used a Delphi questionnaire to collect qualitative data on barriers and facilitators to nurse-led model implementation. Thematic analysis was used to categorize the data into three main components of Michie et al.'s theoretical model: Capability, Opportunity, and Motivation. | The study found that nurse-led EMA provision is supported by respondents, but there are barriers to its implementation that need to be addressed. The study highlights the importance of nurse-led models in improving access to abortion services and overcoming provider shortages. |
| De Zordo (2018) | Italy and Cataluña | 54 obstetricians-gynaecologists at 4 hospitals providing abortion care in Rome and Milan, and with 23 obstetricians-gynaecologists at 2 hospitals and one clinic providing abortion care in Barcelona | This article examines obstetrician-gynecologists' attitudes toward abortion and how they view the embryo/fetus, abortion, and the pregnant woman seeking abortion care. The authors explore the potential role of abortion providers in perpetuating the stigma surrounding abortion. | Descriptive qualitative | Data were collected through intensive fieldwork consisting of short questionnaires and in-depth interviews. No data analysis was described in the published article. | The increasing medicalization of contraception and reproduction has led to the stigmatization of voluntary abortion in a context of declining fertility rates. This explains why obstetrician-gynecologists in Catalan hospitals do not experience abortion stigma and are less opposed to abortion care than their Italian counterparts. |
| Ennis et al. (2023) | Canada | 75 respondents comments of abortion clinicians and administrators. | The authors conducted a exploratory survey aimed to explore stigma ​​and harassment experiences among abortion clinicians and abortion ​​service administrators. | Mixed-Methods Approach, Descriptive qualitative | The researchers conducted a reflexive thematic analysis of open-ended responses from administrators of dedicated abortion services. They independently coded a subset of responses, compared codes, and agreed on an initial codebook. They then divided and coded the remaining responses, engaging in critical reflection and discussion to refine codes and descriptions. The researchers grouped similar codes into interpretive themes and presented their findings in an explanatory narrative. | Clinicians and administrators reported experiencing harassment, with higher-volume abortion providers and administrators facing more harassment, often in the form of picketing. Respondents expressed concerns about harassment from protesters, which they felt negatively impacted patients and led some providers to avoid openly advertising their abortion services. Many respondents desired "bubble zones" to protect themselves and their patients from protesters. Overall, the findings highlight the significant stigma and harassment faced by those involved in abortion care. |
| Fay et al. (2016) | United Kingdom | 10 participants (six men and four women) Maternal–fetal medicine specialist who currently conducted feticides and had performed so for at least a year From a variety of ​ethnicities and religious affiliations | The aim of the study is to explore how maternal-fetal medicine specialists experience and interpret the performance of feticide for fetal anomaly in late termination of pregnancy. | Phenomenology and interpretation | The researchers used a semi-structured interview schedule to elicit personal and professional experiences of conducting feticide. They then used Interpretative Phenomenological Analysis (IPA) to analyze the data, identifying initial and overarching interpretive themes and clustering them within individual transcripts before organizing them into master tables. | The study found that an ongoing doctor-patient relationship facilitated participants' self-image as clinicians when performing feticide. Coping involved rationalization and supportive team relationships. Participants selectively disclosed their involvement in feticide to avoid negative judgment, preferring a lack of public awareness. They also reframed the loss to make it more palatable to others. Personal and professional vulnerability led to selective disclosure among participants. |
| Hasselbacher et al. (2020) | United States, Illinois | 31 participants  Sex: 21 female, 10 male Occupation: obstetrician-gynecologists (n=19), another type of clinician (n=4) (nurse-midwife, family physician or other physician) and non-clinicians (n=8) | The study aims to compare how providers in Catholic, Protestant, and secular health systems experience hospital abortion policies in Illinois, where there is a prevalence of faith-based hospitals and strong legal protections for abortion access. The study seeks to identify key themes related to policy implementation, transparency, and impact on provider experience and patient care. | Descriptive qualitative | The authors used interviews to collect data and a thematic content approach to analyze the data, developing a codebook and coding the transcripts for consistency and concordance. | Religious restrictions in hospitals lead to delays, financial barriers, and stigma for patients seeking abortions, with Protestant hospitals being more flexible in referring patients. Communicating these restrictions can also contribute to stigma and emotional difficulties for patients. |
| Holten et al. (2021) | Netherlands | 200 abortion-seekers’ emails, 20 abortion seekers & 14 health professionals  Age range: 15-35 years | Women's experiences with (online) abortion services and relevant abortion providers' experiences in providing care to identify key barriers for women seeking abortion in the Netherlands. | Grounded theory method, constructivist medical anthropological approach, reproductive justice theoretical framework | Semi-structured in-depth interviews were conducted and analyzed using an abbreviated grounded theory method. In addition, emails from WoW abortion seekers were deductively coded using the codes that emerged from the interviews. | Key findings show that women in the Netherlands lack autonomy in accessing abortion care due to institutionalized taboos, complex regulations, lack of permeability for marginalized groups, and inability to speak openly. To increase autonomy, legislators and policy makers must trust women, avoid stigmatizing abortion, and develop systemic support for vulnerable groups. |
| Homaifer et al. (2017) | United States, Nebraska | 431 clinicians Mean age: (mean±SD), years 46.7±11.6  Sex: Female (n=277, 64%), male (n=154, 36%) Marital status: Single (n=52, 12%), ​married (n=371, 88%)  Children: None (n=44, 12%), ​One or more (n=328, 88%)  Race: White (n=401, 96%), Nonwhite (n=17, 4%) Practice characteristics Specialty  Religion: None (n=28, 7%), ​Protestant (n=251, 60%), ​Catholic (n=128, 31%), ​Other (n=11, 3%)  Intrinsic religiosity: Low (n=180, 44%), ​Moderate (n=98, 24%), ​High (n=130, 32%)  Occupation: Obstetrics and gynecology (n=87, 20%), Family medicine (n=344, 80%) Clinician type Physician (n=204, 47%), Advanced-practice nurse (n=116, 27%), Physician assistant (n=111, 26%)  Years in practice: (mean±SD) 15.9±11.1 | The study focuses on abortion referral practices and motivations, with particular emphasis on rural versus urban clinicians. | Descriptive qualitative | The authors analyzed survey responses thematically and developed codes to describe referral behavior and reasons for referral. They used written comments and multiple-choice responses to assign codes, with one author reviewing and coding all responses and another author flagging discrepancies. | Abortion stigma affects referrals, as some clinicians avoid staff tensions or have moral objections. Some refer women for additional evaluation or counseling before abortion, while others try to dissuade them. Abortion referrals merit heightened privacy concerns because of the existing controversy and stigma. Clinicians fear negative community reaction if abortions or referrals become known. |
| Hulme-Chambers et al. (2018) | Australia, Victoria | 6 training providers for MToP  Sex: all female Occupation: Clinical specialist (n=2), Program executive (n=3), Program manager (n=1)  19 training participants Sex: Female (n=11), male (n=2) Occupation: General practitioner (n=2), General practitioner (n=5), Nurse (n=6) | The study aimed to explore the factors that enabled and challenged decentralization efforts to increase rural MToP service provision in Victoria, Australia. | Descriptive qualitative, conceptual framework of synergies between decentralization and service delivery | The study used individual, semi-structured, in-depth interviews that were digitally recorded and transcribed. Two researchers coded each transcript using an inductive analysis approach to identify themes. A conceptual framework of synergies between decentralization and service delivery was used to understand the study themes. The framework focuses on decision space, institutional capacity, and accountability. The study examined factors that influenced the interactions between decision space, institutional capacity, and accountability as described by both training providers and training participants. | The study found that there is a lack of a national strategy for service delivery and that provider stigma, in the form of negative community or peer attitudes, creates caution among general practitioners and nurses considering service delivery. Challenges to decentralization include the lack of a clear and defined system for MToP provision throughout Victoria, MToP demand versus supply, and provider stigma. Training participants' concerns about the stigma of abortion posed a threat to decentralization, as it could discourage these professionals from providing services. |
| Kavanagh et al. (2018) | United Kingdom, Scotland | 19 participants, nurses (n=9), doctors (n=8) and clinical support workers (n=2) | The study examined the opinions of abortion care providers on the issue of abortion terminology, either in discussions with women or with other professionals. | Descriptive qualitative | The authors used grounded theory qualitative research methodology as the basis for data collection and analysis. Data were collected through in-depth interviews and a self-administered anonymous questionnaire. Constant-comparative methods were used to analyze transcripts and modify the interview schedule. Sociological theories were used to explain the reported use of abortion terminology. | The study found that more respondents found the term "abortion" distressing than "termination of pregnancy. Abortion care providers prefer terminology that effectively manages abortion stigma and is perceived as specific. Participants described "abortion" as harsh and stigmatizing, while "termination of pregnancy" was perceived as softer. Some providers intentionally use "abortion" to emphasize the seriousness of the procedure. |
| Keogh et al. (2017) | Australia, Victoria | 19 individuals with abortion services provision experiences Gender: female (n=15), male (n=4)  Professional role: General practitioner (n=5), Obstetrician/gynaecologist (n=4), Medical practitioner (n=3), Service manager (n=3), Primary health care nurse (n=2), Psychologist (n=1), Sexual health physician (n=1) | To explore the law reform on attitudes toward and availability of abortion with abortion service providers. | Descriptive qualitative | The study used semi-structured interviews and identified four broad themes from the data. They classified, compared, grouped, and refined groupings of text segments within one of the themes. | Key findings of the study highlight concerns about limited public provision of surgical abortion, reduced access to abortion after 20 weeks, persistent stigma, lack of a national strategy for equitable abortion provision, and an unsustainable workforce. Participants agreed that there is still stigma associated with abortion and that access has not improved following law reform. However, there were differing views on whether increasing access and reducing stigma was a core purpose of law reform. |
| Kim et al. (2021) | United States, Ohio | 50 administrators in charge of setting clinical protocols regarding options counseling after a positive pregnancy Race/Sex: Black woman (n=6, 12%) ​, white man (n=5, 10%), white woman (n=39, 78%)  Organizational Type: Primary Care Organization (n=11, 22%), Public Health Department (n=23, 46%), Specialized Reproductive Healthcare Organization (n=8, 16%) | To explore the structural consequences of endorsed stigma among institutional authorities within medical specialties that do not directly provide abortion care. | Descriptive qualitative | The researchers used semi-structured, in-depth interviews and modified grounded theory to analyze data on pregnancy testing and abortion referral practices. They compared their findings to national standards and developed codes and subcodes to identify themes. Consensus was reached among all coders to draw final conclusions about organizational-level practices. | The study found that administrators' misunderstanding of policy and endorsement of abortion stigma shaped safety-net health organizations' response to federal policy, resulting in the structural stigmatization of abortion. Non-referral approaches were often due to administrators' misunderstanding of policy or endorsement of abortion stigma. Institutional entrepreneurship was not effective in reducing the structural stigma of abortion, as evidenced by two specialized reproductive health care organizations being forced to disenroll from the Title X program after providing facilitated referrals. |
| Lee et al. (2023) | United States, Rhode Island, Massachusetts, New Hampshire, Vermont, or Maine | 21 primary care providers (PCPs), self-identified as trained in medication or procedural abortion care, self-identified as desiring to provide abortion, and saw primary care patients  Sex: Female or woman (n=18, 86%) ​Male or man (n=3, 14%)  Race/ethnicity: Asian or Asian American(n=1, 5%), ​Hispanic or Latino white (n=1, 5%) ​White or Caucasian (n=17, 81%), ​Multiracial (n=1, 5%), ​Other (n=1, 5%)  Religious affiliation: Atheist or none (n=15, 71%), ​Buddhist (n=1, 5%), ​Catholic (n=1, 5%), ​Jewish (n=1, 5%), ​​Quaker (n=1, 5%), ​Unitarian Universalist (n=2, 10%)  Self-rated religious importance: Not at all important (n=13, 62%), ​Not very important (n=4, 19%), ​Fairly important (n=1, 5%), ​Very important (n=2, 10%), ​Most important (n=1, 5%) | The study aims to explore the unique barriers that trained PCPs face in integrating abortion into primary care practice in order to inform future interventions. | Descriptive qualitative | The researchers used a semi-structured interview to collect data. They developed a list of codes based on the literature and discussed emerging themes to create a final coding scheme. They coded independently and wrote narrative memos for each thematic area. They stopped recruiting when thematic saturation was reached and used an inductive content analysis approach to synthesize themes and identify patterns. | The study found that logistical barriers to providing abortion services in primary care were driven by stigma and moral opposition, as well as interprofessional tensions with obstetrics and gynecology and radiology departments. Individual opposition from leadership, staff, and peers was a commonly cited barrier, while tensions with other departments delayed or prevented the establishment of abortion services. Successful partnerships with OB/GYNs and appeals to department chairs were identified as strategies for overcoming these barriers. |
| Lindsey et al. (2023) | United States, Georgia | 20 community-based doulas with 8 who provide full spectrum services including abortion. Gender: cisgender female (n=18, 90%), nonbinary/genderqueer (n=2, 10%) Age: Under 25 (n=1, 5%), 25-35 (n=8, 40%), 36-45 (n=7, 35%), 46 or Older (n=4, 20%)  Race/Ethicity: Black or African American (n=9, 45%), White (n=8, 40%), Hispanic or Latinx (n=1, 5%), Multiracial (n=2 10%)  Time as Doula: Less than 1 year (n=2, 10%), 1–3 years (n=9, 45%), More than 3 and up to 9 years (n=5, 25%), More than 9 years (n=4, 20%)  Services: preconception/fertility (7, 35%), prenatal ​(9, 45%), birth (17, 85%), postpartum (12, 60%), abortion (7, 35%), ​full spectrum (8, 40%), radical/justice (4, 20%), and death/grief/ ​loss/bereavement (4, 20%). | This article is co-led by a doula-researcher and a non-profit community-based maternal and child health organization, and involves in-depth interviews and surveys with doulas answering the following questions: 1) How does the ​doula community in metro-Atlanta view doula-supported abortion ​services and abortion generally?; (2) How do abortion doulas ​describe their services?; and (3) What are the facilitators and barriers ​to accessing abortion doula support in metro-Atlanta?. | Descriptive qualitative | The researchers used in-depth interview transcripts, which were cleaned and de-identified, and analyzed them using qualitative software. The research team employed memo-writing techniques to summarize the main content of each interview and identify the top ten emerging topics. They used a semi-deductive coding structure, resulting in both deductive and inductive codes related to various aspects of abortion, contraception, doula work, underserved populations, challenges, client stories, medical outcomes, and the impact of COVID-19. Two team members coded a third of the transcripts together, reached consensus, and then individually coded the remaining transcripts. The coding group developed analytic memos for each code to identify themes within and across the codes, supported by additional analyses within the software. | Abortion doulas are committed to reproductive autonomy, but face challenges due to abortion stigma and restrictive laws. They provide diverse services that benefit clients, but experience stigma from their own communities. Despite this, they are determined to continue supporting reproductive health access, envisioning community-based care that empowers clients. Doulas believe people should have control over their own health and that doulas can help equip them to do so. |
| Mainey et al. (2022) | Australia | 18 nurses and midwives who provided abortion care with first-hand ​experiencing of providing care to people victimized by GBV. | The authors aimedto describe the process by which Australian nurses and midwives provide abortion care to people who have experienced gender-based violence. | Intersectional feminism | The research team, consisting of a graduate student and her two supervisors, closely reviewed the participants' transcripts to develop initial line-by-line and action-by-action codes. The lead author wrote memos and drew diagrams to capture the meaning of the codes, the constant comparative process, and her insights into the data. The team created additional interview questions based on the important and common codes and continued the process until data saturation was reached. | Participants navigated a complex system to provide person-centered abortion care to those affected by gender-based violence. When the system was woman-centered, they were able to achieve positive outcomes. However, when they encountered barriers, they resorted to bending or breaking rules to facilitate timely, holistic care, even when they felt professionally compromised. The grounded theory reveals two cyclical pathways: working with a woman-centered system led to person-centered outcomes, while being backed into a corner due to increasing pregnancy and health/safety risks led to doing the "wrong thing for the right reason" and feeling justified. |
| McLeod et al. (2022) | United States | 106 physicians who had ever provided abortion care | The authors sought to determine the prevalence, risk factors, and nature of contemporary harassment of abortion providers. | Descriptive qualitative | The study used a cross-sectional survey and qualitatively analyzed free-text responses using both deductive and inductive techniques. Two trained coders independently coded the responses and developed a codebook of definitions and examples. The authors identified and distilled overarching themes throughout the process. | Abortion providers face harassment and stigmatization, including invasive and threatening communications, doxing, and property damage. While violent harassment is rare, providers often fear for their safety and that of their loved ones. This isolation from their communities perpetuates stigma and harassment. Providers often take protective measures and avoid practicing in hostile areas. |
| (Rostagnol (2018) | Spain, Andalusia | 15 health care professionals Occupation: two staff members of the Andalusian Health Service (AHS), five healthcare staff members from public health centres in Granada and Seville, four physicians working in certified private clinics from Granada, Seville and Malaga, and four abortion legalization activists from Seville and Granada. | The purpose of this article is to examine the current situation of abortion in Andalusia, with particular attention to the effects of the debates surrounding the Gallardón bill on voluntary abortion. | Feminist theory | This article is based on fieldwork conducted in Andalusia over two periods in 2015 and 2016, including in-depth interviews with gynecologists working in public hospitals and certified private clinics, as well as pro-abortion and feminist activists and Andalusian Health Service staff. | Stigma appeared to be more of a fear than a reality for health professionals in public hospitals. Abortion providers feel they have an important role to play and are supported by training courses and international networks. |
| Ryan et al. (2022) | Republic of Ireland | 7 therapist or counsellor and had experience working with clients experiencing a crisis pregnancy center | The research design focuses on describing the experience of providing CPC in the context of recent legislative changes relating to abortion provision in Ireland. | Interpretative phenomenological analysis | Data were collected through semi-structured interviews and analyzed using the steps outlined by Smith et al. 2009. Four overarching themes emerged from the data, including the challenges counselors faced, their post-referendum experiences, and their personal and professional motivations for engaging in this work. | The study used phenomenological analysis to identify four themes related to crisis pregnancy counseling in Ireland: tensions created by legislation, the impact of societal views and stigma, a new sense of freedom, and finding meaning and motivation in supporting women's choices. The second theme highlighted the burden of social stigma and fear of judgment on counselors, while the third theme focused on the positive impact of the referendum and legislative changes in reducing stigma and increasing acceptance of crisis pregnancy counseling. |
| Singh et al. (2023) | Australia | 18 GPs who provide EMA to women from ​​CALD backgrounds.  Age: 30–39 (n=14), 40–49 (n=3), ≥50 (n=1) Sex: 14 female, 4 male Years providing EMA: <1 (n=1), 1 to <2 (n=5), 2 to <3 (n=4), 3 to <4 (n=3), 4 to <5 (n=2), ≥5 (n=3)  CALD status (self-reported): Non-CALD background (n=8), CALD background (n=10) | The authors' objectives were to explore family physicians' experiences in providing EMA services to women from culturally and linguistically diverse backgrounds and to describe family physicians' perspectives on improving EMA care for this population. | Qualitative–descriptive approach | The researchers transcribed the interview data verbatim and deidentified them. They stored and managed the data using NVivo software, with access restricted to the project researchers. The researchers used Braun and Clarke's six-phase framework to guide the reflexive thematic analysis process. Two researchers generated initial codes by independently coding five transcripts, resolving any discrepancies to create a final coding scheme. One researcher then used this scheme to code the remaining transcripts and group the codes into themes. The researchers reached consensus on the findings and themes, which increased the qualitative rigor and reliability of the analysis. | Access to early medical abortion (EMA) services is challenging for women from culturally and linguistically diverse (CALD) backgrounds due to the stigma associated with abortion within their cultural and/or religious communities, which can create a conflict between their personal needs and their community's expectations. |
| Summit et al. (2020) | United States | 28 physicians  Sex: 22 female, 6 male Primary practice setting: Federally qualified health center (FQHC) (n=9), Family medicine clinic (not FQHC) (n=10), Dedicated abortion clinic (n=4)  Abortion provision since residency: Procedural and medication abortion (n=10), ​Medication abortion only (n=3), ​None (n=15) Abortion provision setting (n=13): Dedicated abortion clinic only (n=3), ​Primary care setting only (n=4), ​Dedicated abortion clinic and primary care setting (n=6) | This qualitative study aims to understand the reasons why abortion-trained physicians in the United States do or do not provide abortion care after completing their residencies. | Descriptive qualitative, social-ecological framework | The study used semi-structured interviews that were recorded and transcribed, with a codebook developed and coded. Thematic saturation was achieved and themes were mapped to levels within the social-ecological framework. The geographic distribution of family medicine residency programs with abortion training is predominantly in the West and Northeast. | The study found that barriers and enablers to abortion provision exist at all levels of the social-ecological model, including legal, institutional, societal, and individual factors. Physicians' social and professional networks were an important factor influencing abortion provision, with supportive staff and colleagues and mentorship being enablers, while unsupportive professional contexts and stigma were barriers. Stigma surrounding the provision of abortion was a significant issue for some providers, particularly those in rural areas and the Midwest. Providers were stigmatized for providing or attempting to provide abortion services, which often made such care impossible. |
| Warren et al. (2022) | United States, Ohio | 10 staff members at eight CPC Age range: 27-71 years Race: White (n=9), ​Multiracial (n=1)  Occupation: executive director (n=7), ultrasound technician (n=1), ​client services manager (n=1), and social media manager (n=1) | Examine how Crisis Pregnancy Centers (CPC) serve as sites where people seeking abortion care are directly stigmatized by the CPC as an institution and by CPC staff as individuals. | Descriptive Qualitative | The study used semi-structured qualitative interviews and field notes to collect data from CPCs. Two phases of coding were conducted, with initial analysis focusing on themes related to why clients choose CPCs and how staff understand their purpose. Secondary analysis revealed new themes related to stigma and health misinformation. | The researchers found enacted and anticipated stigma and documented how women considering abortion mitigate stigma through impression management and other responsive mechanisms. |
