# Supplementary material for: Abortion stigma among abortion providers in high-income countries: a mixed methods systematic review
Source: Sex Reprod Health Matters. 2026 May 22;33(1):2668884. doi: 10.1080/26410397.2026.2668884 (PMC13276811; doi:10.1080/26410397.2026.2668884)
Supplement: Supplementary Table 10. Meta-synthesized findings. [file ZRHM_A_2668884_SM5959.docx]

Supplementary Table 10. Meta-synthesized findings.

| Meta-synthesized finding | Dependability | Credibility | ConQual | Comments |
| --- | --- | --- | --- | --- |
| 1. Individual abortion stigma:  Abortion providers face stigma and discrimination from colleagues, friends, family, and local communities, affecting their practice, finances, and professional growth. They encounter harassment, threats, and social isolation, sometimes leading to forced cessation of practice or reduced service offerings, thus creating access gaps. This stigma stems from cultural and religious norms and the prominent "pro-life" societal stance. Providers grappled with the contrast between their professional identity and public perception, often limiting advertising or selectively disclosing services to avoid a backlash. Those in rural and conservative areas were particularly wary of community reaction (53,57–63,66,67,71,72,74,76–78,80). | Moderate | High | Moderate | - Dependability: Downgraded one level. Majority of studies scored 3/5 for 5 criteria. - Credibility: Not downgraded due to a majority of unequivocal (U) findings. - U=29, C=1 |
| 2. Institutional and structural abortion stigma:  Healthcare providers recognize the stigma and barriers to abortion care, with some noting that conveying religious restrictions can contribute to stigma. Conscientious objections by health care professionals have become obstacles to reproductive rights. Restrictive abortion policies sustain stigma and affect abortion doula services. While some providers downplay the significance of stigma, others have reframed pregnancy loss as more acceptable to others (59,64–66,69,70,72,75,76,78). | Moderate | High | Moderate | - Dependability: Downgraded one level. Majority of studies scored 3/5 for 5 criteria. - Credibility: Not downgraded due to most findings being unequivocal (U). - U=16, C=2 |
| 3. Mitigating factors:  Legislative changes have lessened the stigma and secrecy surrounding abortion counselling, enabling providers to deliver safe, timely, and non-judgmental care. Healthcare providers stress recognizing clients' cultural and religious values to foster trust and rapport. Despite persistent stigma, healthcare providers are dedicated to empowering clients and advocating for their reproductive health, ensuring that they receive accurate information for informed decision making (72,73,76,77,79). | Moderate | High | Moderate | - Dependability: Downgraded one level. Majority of studies scored 3/5 for 5 criteria. - Credibility: Not downgraded due to all findings being unequivocal (U). - U=10, C=0 |
