# Supplementary material for: Abortion stigma among abortion providers in high-income countries: a mixed methods systematic review
Source: Sex Reprod Health Matters. 2026 May 22;33(1):2668884. doi: 10.1080/26410397.2026.2668884 (PMC13276811; doi:10.1080/26410397.2026.2668884)
Supplement: Supplementary Table 4 Critical Appraisal of the qualitative studies [file ZRHM_A_2668884_SM5958.docx]

Supplementary Table 4 Critical Appraisal of the qualitative studies

| Citation | Q1 | Q2 | Q3 | Q4 | Q5 | Q6 | Q7 | Q8 | Q9 | Q10 |
| --- | --- | --- | --- | --- | --- | --- | --- | --- | --- | --- |
| Baier & Behnke (2024) | N | (Y) | Y | Y | Y | Y | Y | Y | Y | Y |
| Chowdhary et al. (2022) | N | Y | Y | Y | Y | N | N | Y | Y | Y |
| Dawson et al. (2017) | N | (Y) | Y | Y | N | N | Y | Y | Y | Y |
| Deb et al. (2020) | N | Y | Y | Y | Y | N | N | Y | Y | Y |
| De Moel-Mandel et al. (2021) | N | (Y) | Y | Y | N | N | N | Y | Y | Y |
| De Zordo (2018) | N | Y | Y | Y | Y | Y | Y | Y | Y | Y |
| Ennis et al. (2023) | N | (Y) | Y | Y | Y | N | N | Y | Y | Y |
| Fay et al. (2016) | N | (Y) | Y | Y | N | N | N | Y | Y | Y |
| Hasselbacher et al. (2020) | N | (Y) | Y | Y | Y | Y | Y | Y | Y | Y |
| Holten et al. (2021) | Y | Y | Y | Y | Y | N | U | Y | Y | Y |
| Homaifar et al. (2017) | N | (Y) | Y | Y | Y | N | N | Y | Y | Y |
| Hulme-Chambers et al. (2018) | N | (Y) | Y | Y | Y | N | N | Y | Y | Y |
| Kavanagh et al. (2018) | N | Y | Y | Y | Y | N | N | Y | Y | Y |
| Keogh et al. (2017) | N | (Y) | Y | Y | Y | N | N | Y | Y | Y |
| Kim et al. (2021) | N | (Y) | Y | Y | Y | Y | N | Y | Y | Y |
| Lee et al. (2023) | N | (Y) | Y | Y | Y | N | N | Y | Y | Y |
| Lindsey et al. (2023) | N | (Y) | Y | Y | Y | N | N | Y | Y | Y |
| Mainey et al. (2022) | N | Y | Y | Y | Y | Y | Y | Y | Y | Y |
| Mcleod et al. (2022) | N | (Y) | Y | Y | Y | N | N | Y | Y | Y |
| Rostagnol (2018) | N | Y | Y | Y | Y | Y | N | Y | N | Y |
| Ryan et al. (2022) | N | Y | Y | Y | Y | Y | Y | Y | N | Y |
| Singh et al. (2023) | N | Y | Y | Y | Y | Y | Y | Y | N | Y |
| Summit et al. (2020) | N | (Y) | Y | Y | Y | N | N | Y | Y | Y |
| Warren et al. (2022) | N | (Y) | Y | Y | Y | N | N | Y | Y | Y |

Y= Yes, (Y) = Descriptive qualitative methodology assigned by reviewers, U = Unclear, NN= Not Needed; JBI critical appraisal checklist for qualitative research

Q1 = Is there congruity between the stated philosophical perspective and the research methodology?

Q2 = Is there congruity between the research methodology and the research question or objectives?

Q3 = Is there congruity between the research methodology and the methods used to collect data?

Q4 = Is there congruity between the research methodology and the representation and analysis of data?

Q5 = Is there congruity between the research methodology and the interpretation of results?

Q6 = Is there a statement locating the researcher culturally or theoretically?

Q7 = Is the influence of the researcher on the research, and vice- versa, addressed?

Q8 = Are participants, and their voices, adequately represented?

Q9 = Is the research ethical according to current criteria or, for recent studies, and is there evidence of ethical approval by an appropriate body?

Q10 = Do the conclusions drawn in the research report flow from the analysis, or interpretation, of the data?
