# Supplementary material for: Abortion stigma among abortion providers in high-income countries: a mixed methods systematic review
Source: Sex Reprod Health Matters. 2026 May 22;33(1):2668884. doi: 10.1080/26410397.2026.2668884 (PMC13276811; doi:10.1080/26410397.2026.2668884)
Supplement: Supplementary Table 8. Findings of the included qualitative studies [file ZRHM_A_2668884_SM5957.docx]

Supplementary Table 8. Findings of the included qualitative studies.

| **Health Care Professionals** | | |
| --- | --- | --- |
| **Study: Baier & Behnke (2023). Barriers to abortion provision: A qualitative study among medical students and gynecologists in Berlin, Germany** | | |
| Finding | Interviewees widely perceived abortion as a tabooed and stigmatized procedure in society in general, but particularly in medicine. (U) |  |
| Illustration | “It’s such a hot potato that no one wants to touch. (…) I experience it myself when I say that I am not an anti-abortion activist (…) that I sometimes get strange looks. Or actually experience hostility”^(p.3)^  “One gynecologist who does not perform abortions (physician 3) described how she was aware of the taboo around abortion even in a hospital where some staff provided them: discussions about who in her team was providing abortions and who was not were held in the “copy room,” not in official team meetings.”^(p.3)^  ““I don’t always dare to talk openly about it with friends, because you don’t know what your friends’ attitudes are.”^(p.3)^ |  |
| **Study: Chowdhary *et al.* (2022). Experiences of women in Ireland who accessed abortion by travelling abroad or by using abortion medication at home: a qualitative study** | |  |
| Finding | A provider shared that she had to tell her children about her work sooner than she’d hoped after protestors picketed at their school. One provider recounted receiving multiple threats. (U) |  |
| Illustration | “I was really upset when my photos were posted publicly. I can’t be confident I’m safe.” ^(p.1353)^ |  |
| Finding | Despite unwavering belief in the necessity and legality of their work, all providers said they occasionally struggled with the discrepancy between how they identify with their work and how they are perceived in the environments within which they live and practice. (U) |  |
| Illustration | “I’m always having to defend what I do. Just because I provide doesn’t mean I’m never uncomfortable. There’s more to me than abortion, but that’s all I’m ever labeled as.” ^(p.1353)^ |  |
| Finding | Voicing frustrations over medical professionals being discriminatory and unsupportive, providers said this limited their practice, financial gains, and development. (C) |  |
| Illustration | “Although abortion work is necessary and challenging, I don’t command the same respect or professionalism. I’m tired of being regarded a lesser physician” ^(p.1353)^ |  |
| Finding | Other providers reflected this through their concerns about the absence of medical professionals in the abortion debate. (U) |  |
| Illustration | “While we’re under attack, there is mostly silence from colleagues. When we’re applying for privileges, physicians on boards aren’t stepping up to help.” ^(p.1353)^ |  |
| **Study: Dawson *et al.* (2017) Medical termination of pregnancy in general practice in Australia: a descriptive-interpretive qualitative study** | | |
| Finding | GP MTOP providers noted that they tended to experience subtle judgemental attitudes from others, including strained collegial relationships with colleagues. (U) |  |
| Illustration | "Oh, even some of my colleagues… are very set in their views. I found that when they found out that I’m doing these things that they have viewed me differently which is a bit depressing” ^(p.5)^ |  |
| Finding | One GP had promoted his services through Family Planning NSW, SRH clinics and Women’s Health  Centres. Some GP providers were wary of others knowing about their services (including other health professionals) because of potential backlash. (U) |  |
| Illustration | "I don’t know how you can ethically advertise it without incurring the wrath of the Christian right  wing” ^(p.5)^ |  |
| Finding | GP abortion providers had also experienced negative social consequences from friends. For instance. (U) |  |
| Illustration | "My friends that has not been good. I’ve actually had a lot of people be quite negative towards me when they find out that I’m doing this. That’s a very big turn off… I was actually more upset by some of my friend’s opinions. A few of my friends found it very difficult to deal with the thought of me doing these and it took me a long time to actually tell them that I was doing it … they responded more negatively than I thought they would.” ^(p.5)^ |  |
| Finding | Another doctor dismissed stigma as a minor issue compared with the practical challenges of provision. (U) |  |
| Illustration | "But I mean that’s not the - the main barrier is actually - yeah, it just wouldn’t work for me from a practical point of view at the moment in terms of - I work for X, I don’t have my own rooms and I have to work within X scope of practice.” ^(p.5)^ |  |
| **Study: De Moel-Mandel *et al.* (2021). Identifying barriers and facilitators of full service nurse-led early medication abortion provision: qualitative findings from a Delphi study** | | |
| Finding | Panellists’ concerns related to the opinions of colleagues, family, friends, and members of conservative communities as well as the fear of negative publicity and personal vilification if PHCRNs were to provide EMA services. These concerns are illustrated in the following Quotes. (U) |  |
| Illustration | “Small town mentality – some nurses would be afraid of community backlash..” ^(p.23)^  “While there is some concern about colleagues’ response and judgement, I think it’s more about community backlash and how friends and family might view them.” ^(p.23)^ |  |
| **Study: Deb *et al.* (2020). Providing medical abortion in general practice. General practitioner insights and tips for future providers** | | |
| Finding | Many participants who had communicated their decision to commence medical abortion delivery in their practice simultaneously restricted advertising of the service. They did this out of fear and stigma. (U) |  |
| Illustration | " [Y]ou don’t want to put yourself at risk of maybe being targeted by any anti-abortion campaigners if you’re too visible." ^(p.333)^ |  |
| **Study: Ennis *et al.* (2023). Experience of stigma and harassment among respondents to the 2019 Canadian abortion provider survey** | |  |
| Finding | Respondents perceived that protesters can negatively impact patients, with one administrator reflecting (U) |  |
| Illustration | “We often hear feedback from patients that the protesters who are present outside the hospital cause feelings of shame and guilt.” ^(p.3)^ |  |
| Finding | As described above, respondents expressed wanting bigger and better bubble zones to mitigate these concerns and experiences, but further noted the issue of the rise of “trolls” harassing abortion providers on social media. (U) |  |
| Illustration | “The harassment/stigma is unnecessary and very upsetting to staff and clients. I think the bubble zone should be MUCH larger. Most times, it just rolls off the staff, but sometimes it becomes distressing when social media trolls begin harassing and name-calling. But, haters gotta hate!” ^(p.3)^ |  |
| Finding | Not providing a service because of stigma from colleagues illustrated how interprofessional stigma, and even discrimination, was a critical issue and interfered with abortion provision for some respondents. One respondent described being “forced out” of practice while another was not able to offer surgical abortions, creating access gaps for their local communities. (U) |  |
| Illustration | “I was forced out of my previous partnership by colleagues because of my outspokenness on sexual and reproductive justice, particularly my opposition to proposed bill 207 in Alberta. That left me without a medical home, cut off from my panel of patients and having to commute to the next closest city to start a new practice. I was the only self-identifying abortion provider in a group of 11 physicians. There ought to be some justice for doctors who do the work, when harassed by colleagues who re-fuse to do the work.” ^(p.3)^ |  |
| **Study: De Zordo (2018). From women’s ‘irresponsibility’ to foetal ‘patienthood’: Obstetricians-gynaecologists’ perspectives on abortion and its stigmatisation in Italy and Cataluña** | |  |
| Finding | Many Italian abortion providers defined it as ‘a dirty job’ that ‘nobody wants to do’, whilst recognising that it was a ‘necessary’ service to prevent illegal and unsafe abortions, alongside its importance for women’s rights and self-determination. (U) |  |
| Illustration | “It surely isn’t a pleasant thing … the act, the procedure … obviously it depends also on the week, but in any case nobody likes it, I think … it always is the termination of a pregnancy, of living cells, I don’t know how to explain … it is not an individual, but it is a potential individual … it does not enrich you performing a termination … it is always tough, for the physician too, but one does it.” ^(p.718)^ |  |
| **Study: Fay *et al.* (2016). Maternal–fetal medicine specialists’ experiences of conducting feticide as part of termination of pregnancy: a qualitative study** | | |
| Finding | Selective disclosure i.e. talking only to trusted individuals about conducting feticide of conducting feticides. (U) |  |
| Illustration | “…the average person in the street has no idea that if you want to terminate a pregnancy for an abnormality over twenty-four weeks you have this procedure…” ^(p.96)^  “I wouldn’t talk about it, only my husband, nobody else […] a lot of people wouldn’t even know that feticide happened […] a lot of people might be quite shocked…” ^(p.97)^  “My partner is anxious that it doesn’t become too widely known […] clearly some people will find the whole thing abhorrent…” ^(p.97)^ |  |
| **Study: Hasselbacher *et al.* (2020). “My Hands Are Tied”: Abortion Restrictions and Providers’ Experiences in Religious and Nonreligious Health Care Systems** | |  |
| Finding | Few providers reflected that communicating the hospital’s religious restrictions could also contribute to stigma regarding abortion. One physician mentioned telling a patient about the need for ethics committee approval in a Protestant hospital. (U) |  |
| Illustration | “It’s a difficult decision, in the first place, to abort a fetus that may not be normal, and then to have someone have to second-guess you and make sure the decision is appropriate may make it more difficult for the patient to process.” ^(p.112)^ |  |
| Finding | An obstetrician-gynecologist at a Protestant hospital commented that a patient whose fetus had a lethal anomaly might be thinking. (U) |  |
| Illustration | “I can’t deal with this. I can’t be living every day, feeling the baby moving around, knowing it’s going to die. It’s too hard on me emotionally, and I just want to get it done now.’ That patient would then be informed that we have to present your case to the ethics committee.” ^(p.112)^ |  |
| **Study: Holten *et al*. (2021). Permeability of abortion care in the Netherlands: a qualitative analysis of women’s experiences, health professional perspectives, and the internet resource of Women on Web** | |  |
| Finding | According to one abortion provider, the (Christian) stigma of abortion as sinful has been institutionalised in the abortion care system in the Netherlands since abortion care is separate from routine health care and is in the penal code. (U) |  |
| Illustration | “[…] The moment they are confronted with an unwanted pregnancy, I think every woman in the Netherlands will immediately feel the stigma. […] will have the idea that they are doing something forbidden. […] they have to take a train for three hours to a separate clinic and there are antiabortion protestors, that’s how out-of-the-ordinary it is.” ^(p.166-167)^  “You can go to any general practitioner or gynecologist for the contraceptive pill, counseling during pregnancy and help with getting pregnant, but finding a pill to terminate the pregnancy suddenly becomes difficult.” ^(p.167)^ |  |
| **Study: Homaifar *et al.* (2017). “She's on her own”: a thematic analysis of clinicians' comments on abortion referral.** | | |
| Finding | While abortion stigma was detected in all categories of referral, it explicitly shaped how five clinicians articulated their referral behavior, as a rural family medicine APN states. (C) |  |
| Illustration | "Patient may not want additional information sent. Patients don't want 'connection' to the clinic providing the service. Because of the stigma, I feel these services are not openly communicated." ^(p.4)^ |  |
| Finding | Clinicians also feared a negative reaction from the community if abortions or abortion referrals were to become known: (U) |  |
| Illustration | "We are such a conservative community that we have to be careful to not attract pro-life activity — we support women however!” ^(p.4)^ |  |
| **Study: Hulme-Chambers *et al.* (2018). Medical termination of pregnancy service delivery in the context of decentralization: social and structural influences** | | |
| Finding | Participants expressed concern in being known as a MToP provider and resultant implications for privacy in rural areas. (U) |  |
| Illustration | “A lot of doctors don’t want to be involved in terminations…even though [town name] is a biggish town, it is small enough that people know who does what, basically. Or it’s easier for people to find out I suppose. So a lot of places, it’s all about the stigma.”^(p.8)^  “I hope that I don’t get backlash from the town. I’m sure it will raise eyebrows. It’ll be interesting to see. I would hope that my professionalism…in my practice wouldn’t - even if there was some of those people that held anti-abortion views, that they would get past that and still continue to see me as a practitioner, even if they were aware that I was providing that”^(p.8)^ |  |
| **Study: Kavanagh *et al.* (2018). ‘Abortion’ or ‘termination of pregnancy’? Views from abortion care providers in Scotland, UK** | | |
| Finding | These descriptors were generally spoken of interchangeably, with ‘harsh’ usually qualified to mean harshly judgemental or stigmatising. ‘Termination of pregnancy’ was almost always described as ‘gentler [than ‘abortion’]’. (U) |  |
| Illustration | "[I prefer using ‘termination of pregnancy’] just be-cause abortion seems like a harsh word. It just feels like a harsh word just because it’s not normalised and because there’s certain stigma around [it].”^(p. 125)^  “I think there are some women who don’t like the term abortion, termination of pregnancy seems like a slightly gentler term to use.” ^(p. 125)^ |  |
| **Study: Keogh *et al.* (2017). Intended and unintended consequences of abortion law reform: perspectives of abortion experts in Victoria, Australia** | | |
| Finding | Section 2. Hope (or intent) of law reform and not achieved. Decrease stigma (for doctors and women). Participants agreed that at present, in Victoria, access had not improved following law  reform – indeed, some felt it had shrunk – and that stigma remains for both women and providers. (U) |  |
| Illustration | “I think the intent hopefully was accessibility, but I don’t think it’s been achieved" ^(p.21)^  “I guess what I hoped though was that it would over the years feed into destigmatising the area, feed into abortion being considered a normal aspect of women’s reproductive health care and fertility choices and be seen as just part of a normal gamut of people trying to be in control of their fertility…. Yes, so I’m hoping that that destigmatisation – I think that will be kind of a slowish process." ^(p.21)^  “The reason I felt that that was done was so that there wasn’t sort of that stigma attached to having an abortion …. But I still believe that there is that, you know sort of that stigma attached to having an abortion, and that’s why a lot of practitioners won’t or don’t, sort of aren’t interested in becoming providers for medical terminations as well." ^(p.21)^  “I still think there’s as much stigma around it. No one wants to say they’re having an abortion or tell people." ^(p.21)^ |  |
| **Study: Kim *et al.* (2021). “We have to respect that option”: The abortion aversion complex in safety-net healthcare organizations.** | | |
| Finding | This amounted to a tacit endorsement of abortion stigma. In this case, institutional entrepreneurship potentially reinforced the structural stigmatization of abortion in the absence of enacted policy. (U) |  |
| Illustration | "This is a day-to-day thing of how we’re allowed to present abortion to clients as an option. I don’t even comment on it. This is real in the day-to-day; there’s war. Have you been seeing this at all? With Title X, we have to have policies that say we can’t—this was new for this grant here—that we can’t refer, and there’s some cloudiness on how we can present it as an option if somebody does come in pregnant. And so we’re very in transition right now with all that.” ^(p.5)^ |  |
| Finding | Here again, administrators drew upon their authority as institutional entrepreneurs to endorse abortion stigma and facilitate the continued structural stigmatization of abortion. (U) |  |
| Illustration | "We can’t do any of that. Like I said, we’re prohibited from doing any of that. We can’t do any referrals. We can’t do any of that. I mean, what we can say is: “This is the area in which you need to look at that, that you’re interested in. We suggest that you see one of our OB people so you could see where you are or if you need some counseling.” But we’re prohibited from doing anything else." ^(p.5)^ |  |
| Finding | Thus, by emphasizing their inability to control providers’ conversations about abortion, administrators were positioned as neither endorsing abortion stigma nor destigmatizing abortion. (U) |  |
| Illustration | "These are your options. You can have termination, you could have this, you have that. But we’re not going to say, “Here are the sites you can go to.” Then we just talk to them about, “Is this a pregnancy you are planning on keeping, and what are your thoughts?” But some of them are shocked. They don’t know what they want at that time. So we do give them the options by saying, you know, there is adoption and there is termination. I’m not in the room with the practitioners, but our protocol is providing information regarding their options. We don’t say, “Hey, here’s some clinics in your area,” no. “ ^(p.6)^ |  |
| **Study: Lee *et al.* (2023). Barriers to abortion provision in primary care in New England, 2019–2020: A qualitative study** | | |
| Finding | PCPs reported fear of opposition or known anti-abortion sentiment that prevented establishment of services. (U) |  |
| Illustration | “I started talking to my division chief, who was supportive but concerned, and she was like, ‘No. The CEO is anti-choice. Let’s wait for him to retire” ^(p. 41)^ |  |
| Finding | Another clinician was particularly concerned about their staff. (U) |  |
| Illustration | ““I’m aways away from getting abortion in our practice as a regular treatment option because much of the support staff…I don’t even tell them I do abortions [outside of our primary care practice] because they’re conservative.” ^(p. 41)^ |  |
| Finding | In line with these concerns, one physician reported backlash from staff after beginning to offer abortion care: (U) |  |
| Illustration | “We tried one [clinical site], and there was sort of a revolt by the staff there. There were some threats to go to the media and threats from protesters.” ^(p. 41)^ |  |
| **Study: Lindsey *et al.* (2023). “I can be pro-abortion and pro-birth”: Opportunities and challenges for full spectrum care among doulas in Georgia** | |  |
| Finding | Abortion ban policies, such Texas’s SB 4 and SB 8 bills and Georgia’s embryonic cardiac activity (estimated 6 weeks) abortion ban following the Supreme Court decision to overturn Roe v. Wade, perpetuate abortion stigma. Several doulas reflected on the impact of restrictive bans on their doula services. (U) |  |
| Illustration | "… with all the legislation that’s being passed … maybe trying to figure out the workarounds and making sure that we don’t get in trouble or sued. How we will be able to help people and not endanger our own selves … I’m really concerned about that. I don’t want to – if we [Georgia] turn into Texas, I don’t want to be sued by some random John walking down the street for $10,000 because of my job.” ^(p.09)^ |  |
| Finding | Georgia also has a history of restrictive abortion bans (e.g., mandatory counseling, mandatory waiting period, and at first a 22-week limit now brought down to the estimated 6 weeks) that impact the way that abortion doulas interact with clients and abortion providers. (U) |  |
| Illustration | “… there are very few providers that could give any information or even would give any information for fear of retribution or backlash on abortion services, especially here in Georgia.” ^(p.09)^ |  |
| Finding | When doulas reflected on the possible stigma perpetrated by the larger doula community, most believed that their doula community was accepting of abortion doulas. However, some like Annie felt there were negative perceptions of abortion doulas. (U) |  |
| Illustration | “Probably not well … because a lot of doulas are, although some doulas are very open, I think a lot of doulas come from like upper middle-class families that are … you know. They just wouldn’t do that in our area.” ^(p.09)^ |  |
| Finding | This stigma was not just felt from the doula community, but also from the participant’s larger community of friends, family, and spiritual leaders. (U) |  |
| Illustration | “I got a phone call from my spiritual teacher, “Oh my gosh, you cannot say that! You cannot say that you’re promoting abortions!”. And I said, I’m not promoting anything! I am saying, if you are in that situation, I am here to help.” ^(p.09)^ |  |
| Finding | Despite stigma felt by abortion doulas, participants described their desire to continue working in the abortion space as both a doula and advocate. Alex described this desire in the context of their work as both an abortion and birth/postpartum doula, (U) |  |
| Illustration | “… there are just too many reasons that abortion care should be accessible. You’re not going to change my mind about that. And I think what really confuses people with me in particular is when I go from saying that abortion care should 10,000% be accessible, and I’m like, oh, yes, but natural birth should also be 10,000% accessible … I can be pro-abortion and also be pro-birth.” ^(p.09-10)^ |  |
| Finding | While this question is asked with the intention of seeing how doulas can best support their clients, some doulas envisioned doula work to fully empower clients to make the decisions necessary for their sexual and reproductive health. (U) |  |
| Illustration | “… people want to take control of their own health. I think people know that doulas are important, and doulas are great, and people also want to take back their own health and we need to think about what are the ways we can equip people to do that.” ^(p.10)^ |  |
| **Study: Mainey *et al.* (2022). Working with or against the system: Nurses' and midwives' process of providing abortion care in the context of gender-based violence in Australia** | |  |
| Finding | Empathizing led to doubling down on their commitment to safe, timely and stigma-free care and putting aside conflicting personal values. (U) |  |
| Illustration | “I grew up Catholic…I'm not comfortable (with abortion) after 12 weeks… one woman in particular… Her partner was quite controlling, and she wasn't telling him that she was pregnant…she got an RU486 off of an online site from India… she couldn't get access to anything here, and she was in a real state…by the time she realized that this RU486 wasn't working, she was  14.5 weeks pregnant, and had a noticeable tummy to her. I found that very challenging, but at the same time, I did everything we could… I could see how destroyed she was, and I know that it's not my place to judge… my compassion side overruled that personal value, and I knew that this woman was going to do whatever she could do to have this termination, so she needed to have it safely. P6.” ^(p.10)^ |  |
| **Study: McLoed *et al.* (2022). Individual abortion providers’ experiences with targeted harassment in the United States** | |  |
| Finding | Respondents often reported harassment that was invasive and violated their personal sense of security. (U) |  |
| Illustration | "These targeting incidents took different forms, including indirect communication such as “threatening phone calls in the middle of the night,” “postcards [sent] to my home,” “tweets, emails harassing me,” and clinic protestors ostentatiously identifying providers by name.” ^(p.44)^  "When I came to my car at the end of the day, I noticed the front door was unlocked. I honestly couldn’t remember if I had left it open…When I sat down in the driver’s seat, I noticed that drawn on the INSIDE of my windshield in the fog was a hanger…I felt really vulnerable and frightened by the event.” ^(p.44)^ |  |
| **Study: Rostagnol (2018). Abortion in Andalusia: Women’s Rights after the Gallardón Bill** | | |
| Finding | According to a gynaecologist I interviewed in Seville, they usually turn to obstetric knowledge to perform abortions. Besides, they lack specific units. (U) |  |
| Illustration | "It is terrible because, since there are no specialized units for patients who undergo elective abortion at public hospitals, those women have to share the same wards as the ones in labour. This situation is bad for both groups.” ^(p.127)^ |  |
| Finding | According to a male gynaecologist I interviewed in Granada, today the majority of young gynaecologists are reluctant to perform abortions and several certified private clinics are having trouble recruiting personnel. Physicians explained this issue as follows. (U) |  |
| Illustration | “Young gynaecologists do not want to get involved in performing abortions because their medical school lecturers are objectors, so they do not encourage abortion practice. In fact, it is most likely that they have even threatened those interested in becoming abortion providers, telling them that if they go on with that idea, they would not successfully complete training as doctors.” ^(p.128)^  “Nowadays, there is no training in pregnancy termination at medical schools; it is not a topic It seems it is not considered part of the medical practices during college education. Shouldn’t be so, but it is.” ^(p.128)^  “You know, one of the reasons why gynaecologists do not want to be abortion providers is the lack of prestige. People want to be admired, especially by their colleagues. If you are an abortion provider you might be appreciated by women, but not by your colleagues.” ^(p.128)^  “The main problem I see is that as there are few gynaecologists who perform abortions, we end up being full-time abortion providers, and nobody wants to do the exact same thing all day long, every single day.” ^(p.128)^ |  |
| Finding | At public hospitals, however, gynaecologists are reluctant to provide an abortion even with an LPT diagnosis. (U) |  |
| Illustration | “Here at the hospital, it is very difficult to find a gynaecologist willing to perform an abortion on a woman in her second trimester because there is, let’s say, a serious problem with the foetus. They are afraid that anatomical-pathological tests on the foetus might not provide definite proof of its incompatibility with extra-uterine life, or might not detect an extremely serious mal-formation. So, they end up excusing themselves, and leave it to the certified private clinic.” ^(p.128)^ |  |
| Finding | A very small percentage of healthcare professionals are willing to be abortion providers; those who  are, are associated with certified private clinics and somehow isolated from the rest of their colleagues. (U) |  |
| Illustration | “You know, we are quite isolated. It’s a good thing that ACAI is a strong association, so we can count on each other.” ^(p.129)^ |  |
| Finding | One thing is certain: since the approval of Law 2/2010, the AHS has continued to handle abortions in the same way it did under the previous one, that is, outsourcing them to certified private clinics. (U) |  |
| Illustration | “We had decided not to change in order to ensure the continuity of the service and to avoid problems at the hospitals, should doctors reject these procedures. At the same time, we were concerned with making it easy for women to terminate their pregnancies: our main interest is to avoid barriers for women. Women do not have to go to their family doctor if they don’t want to, they can go to any doctor. We have paid attention to easing the process of getting a VPT for women who want it.” ^(p.129)^ |  |
| Finding | The case of the pharmacy owner, as well as a case in Galicia related to a hospital’s refusal to perform an abortion, allows us to think that conscientious objection by health professionals became a real obstacle for the actual exercise of sexual and reproductive rights. (U) |  |
| Illustration | “At public hospitals, if healthcare professionals don’t want to be in contact with VPT, they just avoid it. There are many gynaecologists that do not have religious or moral reasons to present conscientious objection, yet they prefer not to do so for other reasons, like they don’t want to be singled out, “Hey, that guy is an abortion provider”. I do not perform abortions either, but I don’t have any problem with it; if I have to, I’ll do it.” ^(p.130-131)^  “There are some important gynaecologists here at the hospital, I mean the elders with an important career behind them, who are lecturers, and they are conservatives, very attached to the Catholic Church. They are against abortion. They’re powerful, so some young people don’t want to have them as enemies, which could mean closing doors in their search for better jobs. So, they just avoid abortion care, and that’s it.” ^(p. 131)^  “Some people think that only certified private clinics should perform abortions. Actually that makes it easier for everybody.” ^(p. 131)^  “When one of my patients comes requesting an abortion, I directly send her to a clinic where I know she will get the best possible care. I know the gynaecologists who work over there, they are my friends; they know how to do it much better than we do here at the hospital. But I have to tell you, most of my colleagues at the hospital don’t think as I do.” ^(p. 131)^ |  |
| **Study: Ryan *et al.* (2022). Lifting the cloak of secrecy: Experiences of providing crisis pregnancy counselling in a changing legislative context in Ireland** | | |
| Finding | Feelings of isolation and social disconnect were common among counsellors, with participant A stating that she felt as though she was being hidden from society: (U) |  |
| Illustration | "Prior to the referendum, there was an isolated element to it… my office was in the basement and there was no window, so you know, maybe we weren't hidden down there but there was that sort of metaphorical, you know, keep us hidden." ^(p.25)^ |  |
| Finding | This was attributed to the assumption that they are accepting of abortion as an act of murder, as reflected in the ‘pro-life’ stance prominent in Irish discourse (Hanschmidt et al., 2016; Smyth, 2015). (U) |  |
| Illustration | "I wouldn't tell a whole lot of people, like if anybody knew I was doing that kind of work you know they might have a judgement about oh my god I can't believe you give people information and they're going to kill their baby, how can you live with yourself kind of thing." ^(p.26)^ |  |
| Finding | The anxiety elicited by societal disapproval of abortion was evident as participants spoke about the fear created by protests and demonstrations held by campaigners outside workplaces; (U) |  |
| Illustration | "participant C reported that she knew of ‘colleagues where there was demonstrations outside of the buildings during the time of the referendum’, and participant A stated that ‘there was that fear, you know we had a couple of nasty people do nasty stuff in front of our office…there was I would say a kind of angst’" ^(p.26)^ |  |
| Finding | Participant G further acknowledged the impact that the socio-political attitudes towards abortion had on their work, saying: (U) |  |
| Illustration | "We did have to work within that broader context of where political and social views were much more prominent within the therapy room I suppose at that time… the political piece came into the therapy room if you like, so it definitely did have an impact." ^(p.26)^ |  |
| Finding | The fear of judgement regarding the nature of their work was also felt by participants when interacting with other counsellors within and outside of the CPC network (U) |  |
| Illustration | "Participant G said that due to ‘living in society at that time, where [crisis pregnancy] was quite secretive’, she felt that even when speaking to other professionals, she would wonder ‘who would you say it to or not that I provide Crisis Pregnancy Counselling and I give information on terminations’, as not all counsellors involved in CPC worked in ‘three option’ centres where information on abortion services abroad was provided.”  ^(p.26)^  "There would be a reluctance to kind of name, that yes we did provide information on three options so there was always a caution about it probably. It wouldn't have stopped me saying it necessarily but there would have been a caution, a caution around it and a carefulness I suppose.” ^(p.26)^ |  |
| Finding | This theme reveals the impact on counsellors of being seen to represent a phenomenon long-considered socially unacceptable (U) |  |
| Illustration | "We did have to work within that broader context of where political and social views were much more prominent within the therapy room I suppose at that time… the political piece came into the therapy room if you like, so it definitely did have an impact." ^(p.26)^  "The issue was definitely seen as taboo and I definitely saw this impacting on colleagues, some of them really struggled.” ^(p.26)^ |  |
| Finding | Participant A reported that legislative changes removed a layer of fear and apprehension from the work, as the public perception of CPC as ‘dirty work’ (Hughes, 1951) may be changing and bringing a new sense of acceptance and tolerance for their work (U) |  |
| Illustration | "We did have to work within that broader context of where political and social views were much more prominent within the therapy room I suppose at that time… the political piece came into the therapy room if you like, so it definitely did have an impact." ^(p.26)^ |  |
| Finding | This social shift was understood by counsellors to lessen the burden of shame and stigma carried by clients accessing the service. (U) |  |
| Illustration | "Some of my colleagues have had sting operations, they've had bogus clients, there were rogue organisations, so I think the fact that all those elements will be removed from the landscape will probably be a breath of fresh air and everybody will be more comfortable." ^(p.28)^  "It just feels freer, it feels less restrictive and I think it's more respectful of a woman's choice, its empowering a woman to feel that she can freely make a choice that she's free under the legal, you know, umbrella, so it does shift something.”  ^(p.28)^  “they've got validation from the whole country in lots of ways because it [the referendum] has been passed so there's validation there going look most people are saying absolutely you should have this choice and there's no shame in this choice.”  ^(p.28)^ |  |
| Finding | This, in turn, was felt to change the experience of the work for counsellors, removing a layer of secrecy from the work that was created by societal stigma. (U) |  |
| Illustration | "people will be more open about it maybe now because there is not so much a kind of a shame attached to people having it and the secrecy around it’." ^(p.28)^ |  |
| Finding | Participant D further emphasised that counsellors no longer felt that they were complicit in a secretive or shameful interaction with clients: (U) |  |
| Illustration | "It's [abortion provision] all part of our own system and very normalised whereas before it was like I'm in on some secret with them…I don't feel as much pressure I suppose to alleviate the stigma they might be feeling." ^(p.28)^ |  |
| **Study: Singh *et al.* (2023). General practitioner experiences in delivering early medical abortion services to women from culturally and linguistically diverse backgrounds** | |  |
| Finding | Cultural and/or religious norms that GPs found contributed to abortion being viewed as a taboo subject included the unacceptability of premarital sex, the belief that abortion is morally wrong and the idea that motherhood is central to the construction of a married woman’s identity. (U) |  |
| Illustration | "These values resulted in some women believing that ‘they will be punished by their God’ (GP8, female, Vic) or that they are a ‘bad woman because they are doing an abortion’ (GP9, female, Vic), which could negatively affect emotional wellbeing.” ^(p.560)^ |  |
| Finding | Many GPs discussed that taking the time during consultations to acknowledge the woman’s cultural and/or religious values and views on unintended pregnancy and abortion was necessary to build rapport, inspire trust and provide optimal care: (U) |  |
| Illustration | "It makes things a lot more acceptable for them if they know that you respect or know where they’re coming from with their background needs.” ^(p.560)^ |  |
| **Study: Summit *et al.* (2020). Barriers to and Enablers of Abortion Provision for Family Physicians Trained in Abortion During Residency** | | |
| Finding | Other respondents described providers being stigmatized for offering or attempting to offer abortion services. One physician who had a rotation that took her from the Northwestern city where her residency program was based to a more rural area in the same state recalled. (U) |  |
| Illustration | "When I went to [town], one provider had really been wanting to offer abortion services.… And there had definitely been a demand for them, and…she tried to do it, and…there was a lot of pushback and a lot of picketing." ^(p.155)^ |  |
| Finding | Another respondent described trying to decide whether she could offer abortions in her practice in a small, conservative town in the West. Although she took the advice of an established physician in the community and opted not to provide abortions, she still felt conflicted about her decision. (U) |  |
| Illustration | “I asked an OB doc in that town, ‘Is [abortion care] some-thing that I should try to push to make happen, …or is that professional suicide?’ And…he said, ‘There’s no woman that’s not going to be able to get an abortion because you don’t do abortion care.… Is it worth risking your life, your family’s life, or your job and your paycheck to be able to provide a handful of abortions in your clinic?’ At first, I was like, ‘Well, he’s got a point,’ and he does, but now I’m also pissed at him because he’s…actually wrong. People do have significant hardships and don’t always get access to  the abortion that they want…because their doctor won’t provide it or won’t even have that conversation.” ^(p.155-156)^ |  |
| **Study: Warren et al. (2022). “I felt like I was a bad person… which I’m not”: Stigmatization in crisis pregnancy centers** | |  |
| Finding | In order to facilitate a sense of trust with clients, staff members emphasized the need for a judgment-free zone. (U) |  |
| Illustration | "So, our goal is to just not be like that judgement person but kinda just give them the facts then warm them up." ^(p.4)^ |  |
| Finding | She understood her role as providing clients with correct information and cast herself as a concerned medical professional who wanted to make sure clients have all the salient information: (U) |  |
| Illustration | “That's, again, kind of from my medical and analytical, is that I compare this to any other medical procedure or facility, is any doctor, or nurse, or anyone who wants to perform a surgery or procedure on you, you have to have informed consent. That includes all the risks, all the details of the procedure. You need to sign for that saying, "I fully understand," and they have to make sure that you have the option to understand all your options. If they're not marking body parts that they're going to be doing surgery on, they can get fined and shut down. So, why is it any different for us when we say, "Okay, we need to show you that yes, there is a baby, there is a fetus in your uterus, or no that fetus has passed.” ^(p.4)^ |  |

*C= credible, U= unequivocal*
