# Supplementary material for: Abortion stigma among abortion providers in high-income countries: a mixed methods systematic review
Source: Sex Reprod Health Matters. 2026 May 22;33(1):2668884. doi: 10.1080/26410397.2026.2668884 (PMC13276811; doi:10.1080/26410397.2026.2668884)
Supplement: Supplementary Table 2. Search string [file ZRHM_A_2668884_SM5956.docx]

Supplementary Table 2. Search string

| Search | Query |
| --- | --- |
| #1 Medline (EBSCOhost) | [abortion[tiab] OR pregnancy termination[tiab] OR voluntary pregnancy interruption[tiab]] AND [stigma*[tiab] OR discriminat*[tiab]]* |
| #2 CINHAL (EBSCOhost) | [abortion[tiab] OR pregnancy termination[tiab] OR voluntary pregnancy interruption[tiab]] AND [stigma*[tiab] OR discriminat*[tiab]]* |
| #3PsychINFO (EBSCOhost) | [abortion[tiab] OR pregnancy termination[tiab] OR voluntary pregnancy interruption[tiab]] AND [stigma*[tiab] OR discriminat*[tiab]]* |
| #4 Cochrane | ("abortion" OR "pregnancy termination" AND "discriminat*" OR "stigma*") with Cochrane Library publication date Between Jan 2015 and Jan 2023, in Cochrane Reviews, Trials with 'Public Health', 'Cochrane Germany', 'Pregnancy and Childbirth', 'Gynaecology and Fertility', 'Cochrane Nordic', 'Consumers and Communication', 'Cochrane UK', 'Cochrane Australia', 'Effective Practice and Organisation of Care', 'Cochrane Canada' in Cochrane Groups (Word variations have been searched) |
| #5 LIVIVO | ((abortion OR pregnancy termination OR voluntary pregnancy interruption) AND (discriminat* OR stigma*)) AND PY=2015:2023 |

*Limited to 2015 to 2023 (updated on February 27, 2024)
