# Supplementary material for: Abortion stigma among abortion providers in high-income countries: a mixed methods systematic review
Source: Sex Reprod Health Matters. 2026 May 22;33(1):2668884. doi: 10.1080/26410397.2026.2668884 (PMC13276811; doi:10.1080/26410397.2026.2668884)
Supplement: Supplementary Table 6. Abortion stigma definitions [file ZRHM_A_2668884_SM5955.docx]

Supplementary Table 6. Abortion stigma definitions

| Citation | Explicitly stated self-  definition | Harris et al. (2013) | Kumar, Hessini and Mitchell (2009) | Norris et al. (2011) | O’Donell et al. (2018) | Generic stigma  definition | Not  explicitly stated |
| --- | --- | --- | --- | --- | --- | --- | --- |
| Quantitative Studies | | | | | | | |
| Dempsey et al. (2021) |  | 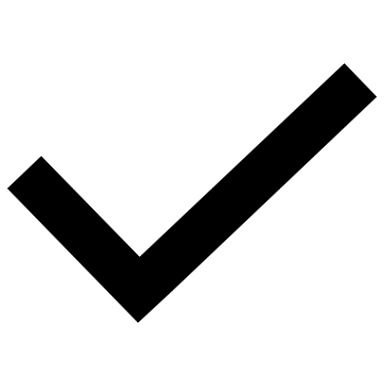 |  | 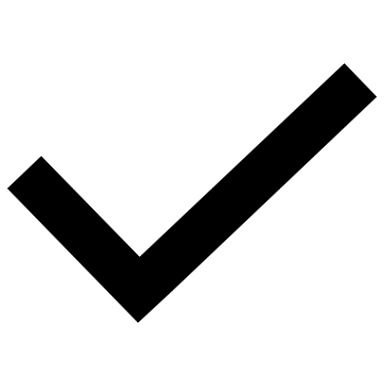 | 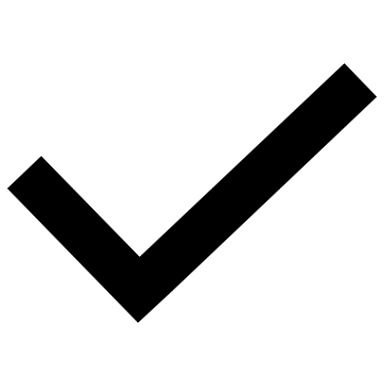 |  |  |
| Haas et al. (2022) |  |  |  |  |  |  | 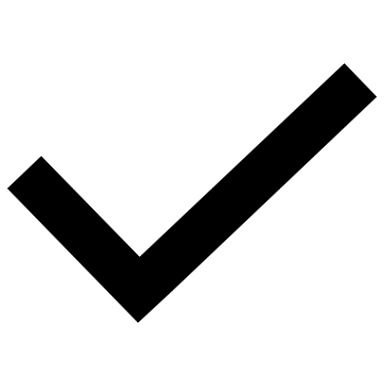 |
| Janiak et al. (2018) |  | 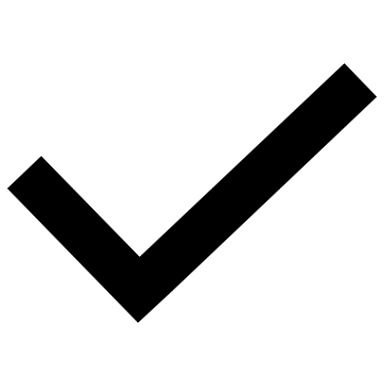 | 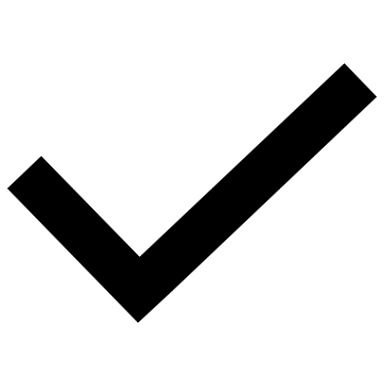 |  | 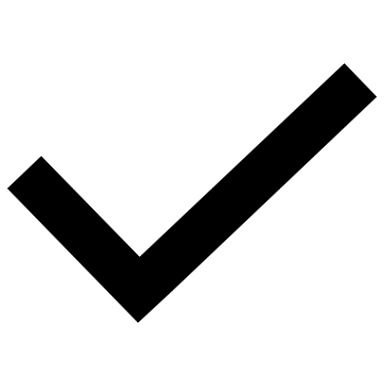 |  |  |
| Martin et al. (2018) |  |  | 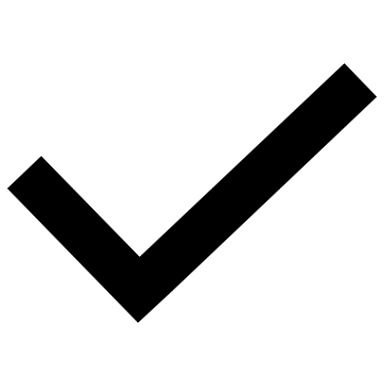 | 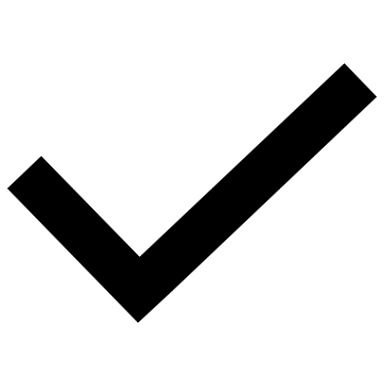 |  |  |  |
| Qualitative Studies | | | | | | | |
| Baier & Behnke (2024) |  |  |  |  |  |  | 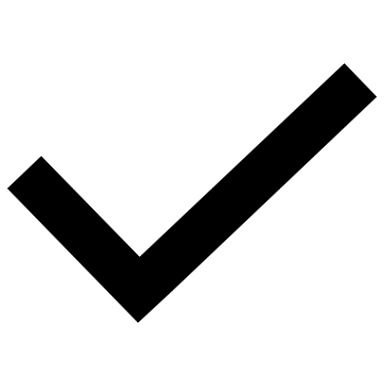 |
| Chowdhary et al. (2022) |  |  |  |  |  |  | 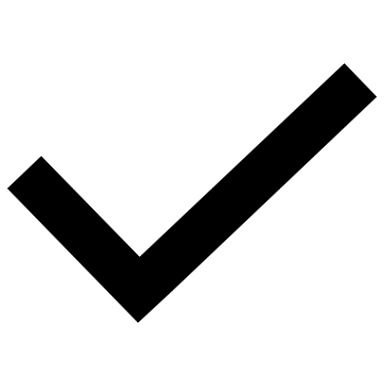 |
| Dawson et al. (2017) |  |  |  |  |  |  | 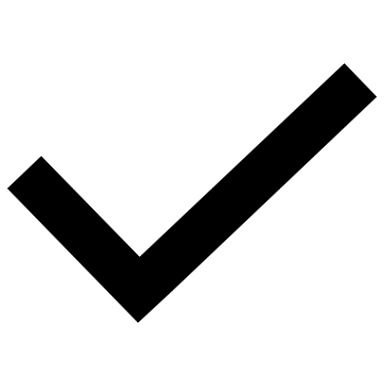 |
| Deb et al. (2020) |  |  |  |  |  |  | 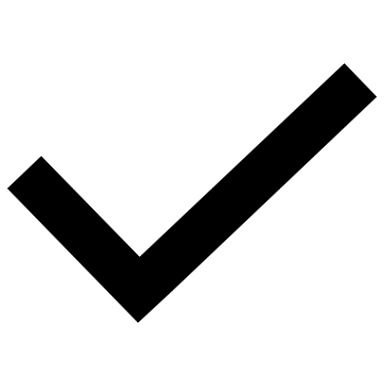 |
| De Moel-Mandel et al. (2021) |  |  |  |  |  |  | 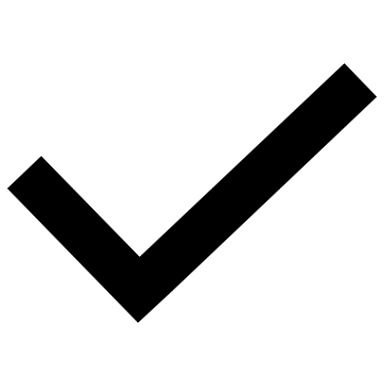 |
| De Zordo (2018) |  |  |  |  |  |  | 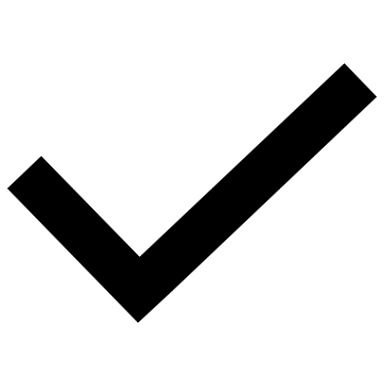 |
| Ennis et al. (2023) |  |  |  |  |  |  | 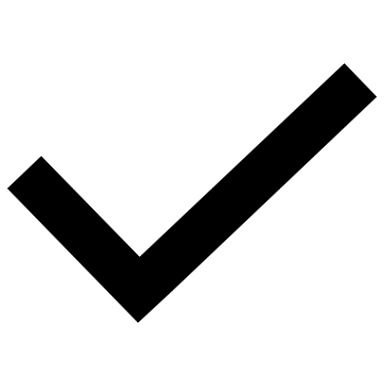 |
| Fay et al. (2016) |  | 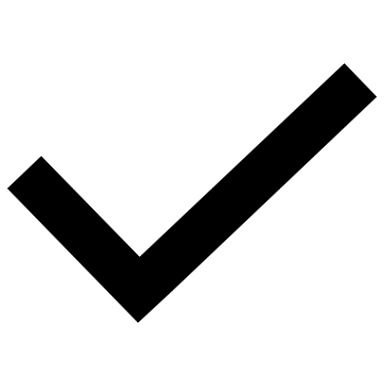 |  |  |  |  |  |
| Hasselbacher et al. (2020) |  |  |  |  |  |  | 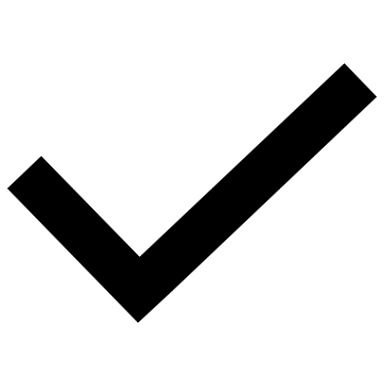 |
| Holten et al. (2021) |  |  |  |  |  |  | 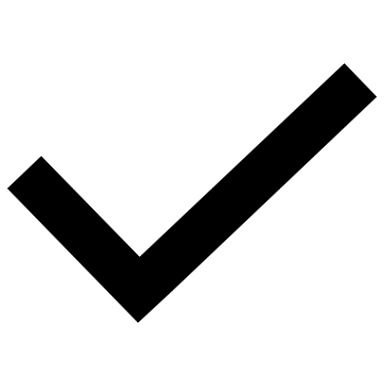 |
| Homaifar et al. (2017) |  |  |  |  |  |  | 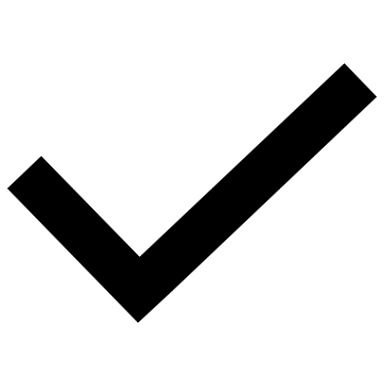 |
| Hulme-Chambers et al. (2018) |  |  | 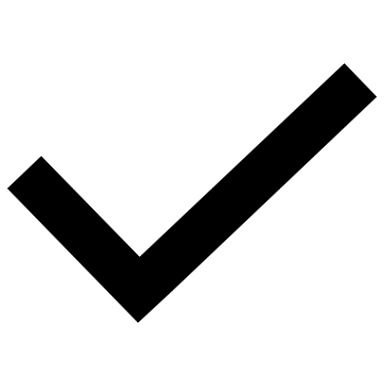 |  |  |  |  |
| Kavanagh et al. (2018) |  |  |  |  |  |  | 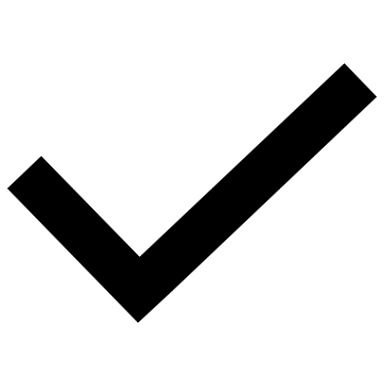 |
| Keogh et al. (2017) |  |  |  |  |  |  | 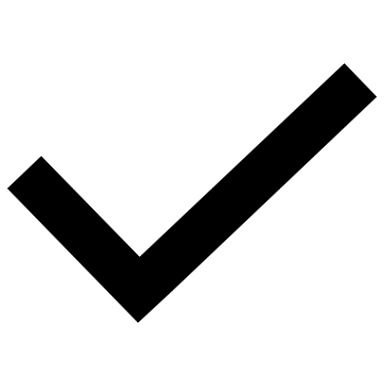 |
| Kim et al. (2021) |  |  | 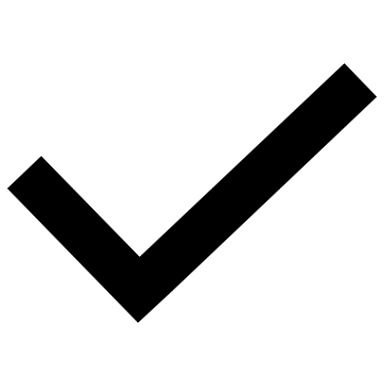 | 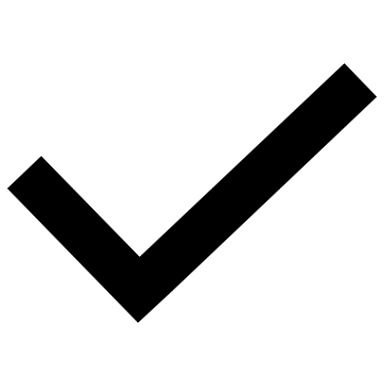 |  |  |  |
| Lee et al. (2023) |  |  |  |  |  |  | 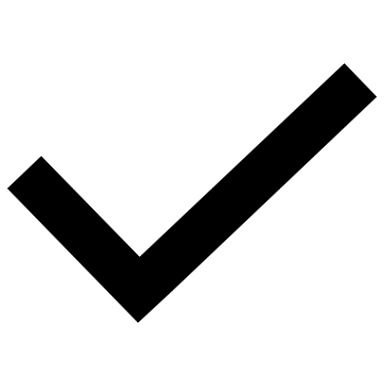 |
| Lindsey et al. (2023) |  |  |  |  |  |  | 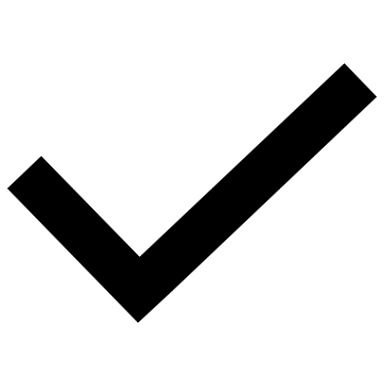 |
| Mainey et al. (2022) |  |  |  |  |  |  | 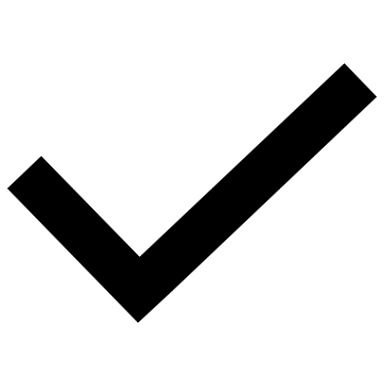 |
| Mcleod et al. (2022) |  | 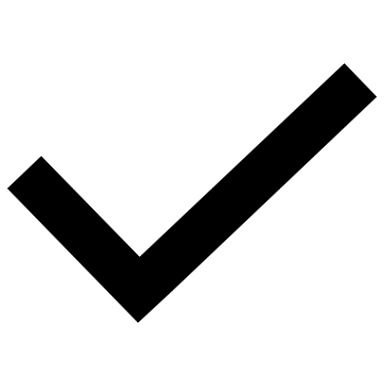 |  |  |  |  |  |
| Rostagnol (2018) |  |  | 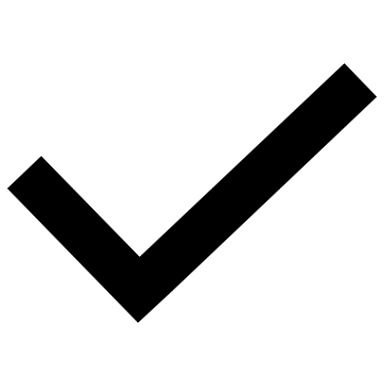 | 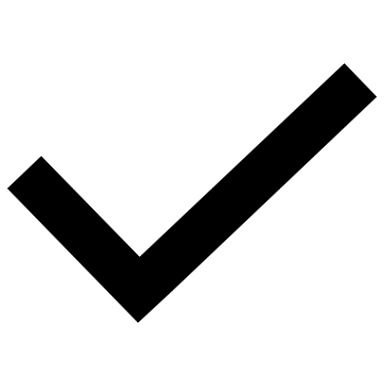 |  |  |  |
| Ryan et al. (2022) |  |  | 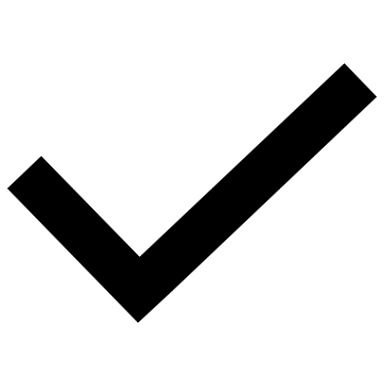 |  | 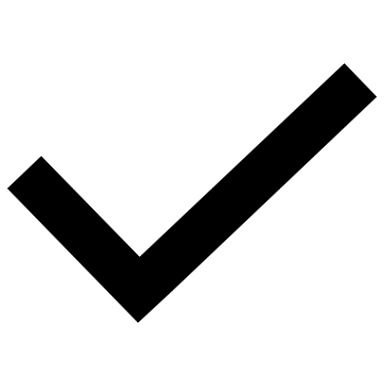 |  |  |
| Singh et al. (2023) |  |  |  |  |  |  | 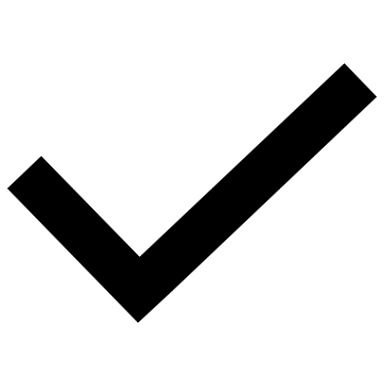 |
| Summit et al. (2020) |  |  |  |  |  |  | 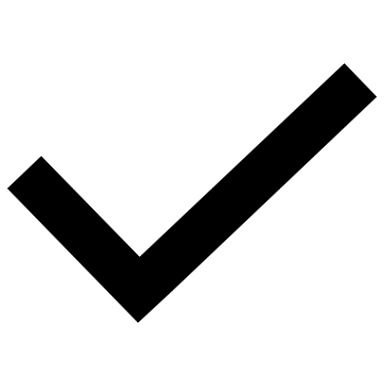 |
| Warren et al. (2022) |  |  |  |  |  | 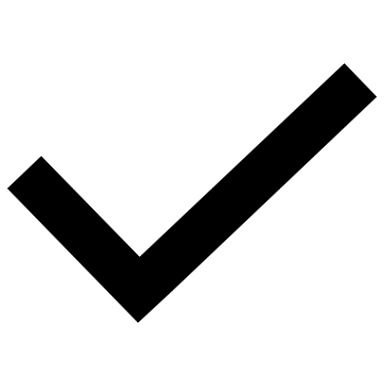 |  |
