# Supplementary material for: Abortion stigma among abortion providers in high-income countries: a mixed methods systematic review
Source: Sex Reprod Health Matters. 2026 May 22;33(1):2668884. doi: 10.1080/26410397.2026.2668884 (PMC13276811; doi:10.1080/26410397.2026.2668884)
Supplement: Supplementary Table 9. Synthesized Qualitative Findings [file ZRHM_A_2668884_SM5954.docx]

Supplementary Table 9. Synthesized Qualitative Findings.

| **Findings** | **Categories** | **Synthesized findings** |
| --- | --- | --- |
| **Synthesized Finding 2 – Individual abortion stigma** |  | Abortion providers face stigma and discrimination from colleagues, friends, family, and local communities, affecting their practice, finances, and professional growth. They encounter harassment, threats, and social isolation, sometimes leading to forced cessation of practice or reduced service offerings, thus creating access gaps. This stigma stems from cultural and religious norms and the prominent "pro-life" societal stance. Providers grappled with the contrast between their professional identity and public perception, often limiting advertising or selectively disclosing services to avoid a backlash. Those in rural and conservative areas were particularly wary of community reaction |
| A provider shared that she had to tell her children about her work sooner than she’d hoped after protestors picketed at their school. One provider recounted receiving multiple threats. (U) | **Perceived stigma by protestors** |  |
| As described above, respondents expressed wanting bigger and better bubble zones to mitigate these concerns and experiences, but further noted the issue of the rise of “trolls” harassing abortion providers on social media. (U) |  |  |
| Respondents often reported harassment that was invasive and violated their personal sense of security. (U) |  |  |
| The anxiety elicited by societal disapproval of abortion was evident as participants spoke about the fear created by protests and demonstrations held by campaigners outside workplaces. (U) |  |  |
| Voicing frustrations over medical professionals being discriminatory and unsupportive, providers said this limited their practice, financial gains, and development. (C) | **Perceived stigma by colleagues** |  |
| GP MTOP providers noted that they tended to experience subtle judgemental attitudes from others, including strained collegial relationships with colleagues. (U) |  |  |
| Not providing a service because of stigma from colleagues illustrated how interprofessional stigma, and even discrimination, was a critical issue and interfered with abortion provision for some respondents. One respondent described being “forced out” of practice while another was not able to offer surgical abortions, creating access gaps for their local communities. (U) |  |  |
| In line with these concerns, one physician reported backlash from staff after beginning to offer abortion care: (U) |  |  |
| GP abortion providers had also experienced negative social consequences from friends. For instance. (U) | **Perceived stigma by social environment** |  |
| This stigma was not just felt from the doula community, but also from the participant’s larger community of friends, family, and spiritual leaders. (U) |  |  |
| Participant G further acknowledged the impact that the socio-political attitudes towards abortion had on their work. (U) | **Perceived stigma by society** |  |
| Interviewees widely perceived abortion as a tabooed and stigmatized procedure in society in general, but particularly in medicine. (U) |  |  |
| Feelings of isolation and social disconnect were common among counsellors, with participant A stating that she felt as though she was being hidden from society: (U) |  |  |
| Cultural and/or religious norms that GPs found contributed to abortion being viewed as a taboo subject included the unacceptability of premarital sex, the belief that abortion is morally wrong and the idea that motherhood is central to the construction of a married woman’s identity. (U) |  |  |
| Another respondent described trying to decide whether she could offer abortions in her practice in a small, conservative town in the West. Although she took the advice of an established physician in the community and opted not to provide abortions, she still felt conflicted about her decision. (U) |  |  |
| This was attributed to the assumption that they are accepting of abortion as an act of murder, as reflected in the ‘pro-life’ stance prominent in Irish discourse. (U) | **Anticipated abortion stigma** |  |
| Other providers reflected this through their concerns about the absence of medical professionals in the abortion debate. (U) |  |  |
| One GP had promoted his services through Family Planning NSW, SRH clinics and Women’s Health Centres. Some GP providers were wary of others knowing about their services (including other health professionals) because of potential backlash. (U) |  |  |
| Panellists’ concerns related to the opinions of colleagues, family, friends, and members of conservative communities as well as the fear of negative publicity and personal vilification if PHCRNs were to provide EMA services. (U) |  |  |
| Many participants who had communicated their decision to commence medical abortion delivery in their practice simultaneously restricted advertising of the service. They did this out of fear and stigma. (U) |  |  |
| Selective disclosure i.e. talking only to trusted individuals about conducting feticide of conducting feticides. (U) |  |  |
| Clinicians also feared a negative reaction from the community if abortions or abortion referrals were to become known. (U) |  |  |
| Participants expressed concern in being known as a MToP provider and resultant implications for privacy in rural areas. (U) |  |  |
| PCPs reported fear of opposition or known anti-abortion sentiment that prevented establishment of services. (U) |  |  |
| Another clinician was particularly concerned about their staff. (U) |  |  |
| When doulas reflected on the possible stigma perpetrated by the larger doula community, most believed that their doula community was accepting of abortion doulas. However, some like Annie felt there were negative perceptions of abortion doulas. (U) |  |  |
| The fear of judgement regarding the nature of their work was also felt by participants when interacting with other counsellors within and outside of the CPC network. (U) |  |  |
| **Synthesized finding 2 – Institutional and structural abortion stigma** |  |  |
| Few providers reflected that communicating the hospital’s religious restrictions could also contribute to stigma regarding abortion. One physician mentioned telling a patient about the need for ethics committee approval in a Protestant hospital. (U) | **Anti-abortion attitudes within the working space** | Healthcare providers recognize the stigma and barriers to abortion care, with some noting that conveying religious restrictions can contribute to stigma. Conscientious objections by health care professionals have become obstacles to reproductive rights. Restrictive abortion policies sustain stigma and affect abortion doula services. While some providers downplay the significance of stigma, others have reframed pregnancy loss as more acceptable to others. |
| An obstetrician-gynecologist at a Protestant hospital commented that a patient whose fetus had a lethal anomaly might be thinking. (C) |  |  |
| According to one abortion provider, the (Christian) stigma of abortion as sinful has been institutionalised in the abortion care system in the Netherlands since abortion care is separate from routine health care and is in the penal code. (U) |  |  |
| According to a gynaecologist I interviewed in Seville, they usually turn to obstetric knowledge to perform abortions. Besides, they lack specific units. (U) |  |  |
| This amounted to a tacit endorsement of abortion stigma. In this case, institutional entrepreneurship potentially reinforced the structural stigmatization of abortion in the absence of enacted policy. (U) |  |  |
| Here again, administrators drew upon their authority as institutional entrepreneurs to endorse abortion stigma and facilitate the continued structural stigmatization of abortion. (U) |  |  |
| Another doctor dismissed stigma as a minor issue compared with the practical challenges of provision. (U) |  |  |
| Other respondents described providers being stigmatized for offering or attempting to offer abortion services. One physician who had a rotation that took her from the Northwestern city where her residency program was based to a more rural area in the same state recalled. (U) |  |  |
| According to a male gynaecologist I interviewed in Granada, today the majority of young gynaecologists are reluctant to perform abortions and several certified private clinics are having trouble recruiting personnel. Physicians explained this issue as follows. (U) |  |  |
| At public hospitals, however, gynaecologists are reluctant to provide an abortion even with an LPT diagnosis. (U) |  |  |
| A very small percentage of healthcare professionals are willing to be abortion providers; those who are, are associated with certified private clinics and somehow isolated from the rest of their colleagues. (U) |  |  |
| The case of the pharmacy owner, as well as a case in Galicia related to a hospital’s refusal to perform an abortion, allows us to think that conscientious objection by health professionals became a real obstacle for the actual exercise of sexual and reproductive rights. (U) |  |  |
| While abortion stigma was detected in all categories of referral, it explicitly shaped how five clinicians articulated their referral behavior, as a rural family medicine APN states. (C) |  |  |
| Section 2. Hope (or intent) of law reform and not achieved. Decrease stigma (for doctors and women). Participants agreed that at present, in Victoria, access had not improved following law reform – indeed, some felt it had shrunk – and that stigma remains for both women and providers. (U) | **Anti-abortion politics** |  |
| Abortion ban policies, such Texas’s SB 4 and SB 8 bills and Georgia’s embryonic cardiac activity (estimated 6 weeks) abortion ban following the Supreme Court decision to overturn Roe v. Wade, perpetuate abortion stigma. Several doulas reflected on the impact of restrictive bans on their doula services. (U) |  |  |
| Georgia also has a history of restrictive abortion bans (e.g., mandatory counseling, mandatory waiting period, and at first a 22-week limit now brought down to the estimated 6 weeks) that impact the way that abortion doulas interact with clients and abortion providers. (U) |  |  |
| One thing is certain: since the approval of Law 2/2010, the AHS has continued to handle abortions in the same way it did under the previous one, that is, outsourcing them to certified private clinics. (U) |  |  |
| This theme reveals the impact on counsellors of being seen to represent a phenomenon long considered socially unacceptable (Ryan 2020 Lifting the cloak.) (U) |  |  |
| **Synthesized Finding 3 – Mitigating factors** | | |
| Despite stigma felt by abortion doulas, participants described their desire to continue working in the abortion space as both a doula and advocate. Alex described this desire in the context of their work as both an abortion and birth/postpartum doula. (U) | **Empowerment of clients** | Legislative changes have lessened the stigma and secrecy surrounding abortion counselling, enabling providers to deliver safe, timely, and non-judgmental care. Healthcare providers stress recognizing clients' cultural and religious values to foster trust and rapport. Despite persistent stigma, healthcare providers are dedicated to empowering clients and advocating for their reproductive health, ensuring that they receive accurate information for informed decision making. |
| While this question is asked with the intention of seeing how doulas can best support their clients, some doulas envisioned doula work to fully empower clients to make the decisions necessary for their sexual and reproductive health. (U) |  |  |
| She understood her role as providing clients with correct information and cast herself as a concerned medical professional who wanted to make sure clients have all the salient information. (U) |  |  |
| Empathizing led to doubling down on their commitment to safe, timely and stigma-free care and putting aside conflicting personal values. (U) | **Judgement free and trust** |  |
| Many GPs discussed that taking the time during consultations to acknowledge the woman’s cultural and/or religious values and views on unintended pregnancy and abortion was necessary to build rapport, inspire trust and provide optimal care. (U) |  |  |
| In order to facilitate a sense of trust with clients, staff members emphasized the need for a judgment-free zone. (U) |  |  |
| Participant A reported that legislative changes removed a layer of fear and apprehension from the work, as the public perception of CPC as ‘dirty work’ (Hughes, 1951) may be changing and bringing a new sense of acceptance and tolerance for their work (U) | **Social shift and legal changes** |  |
| This social shift was understood by counsellors to lessen the burden of shame and stigma carried by clients accessing the service. (U) |  |  |
| This, in turn, was felt to change the experience of the work for counsellors, removing a layer of secrecy from the work that was created by societal stigma. (U) |  |  |
| Participant D further emphasised that counsellors no longer felt that they were complicit in a secretive or shameful interaction with clients. (U) |  |  |
